# Applying Machine Learning to Ultrafast Shape Recognition in Ligand-Based Virtual Screening

**DOI:** 10.3389/fphar.2019.01675

**Published:** 2020-02-19

**Authors:** Etienne Bonanno, Jean-Paul Ebejer

**Affiliations:** ^1^Department of Artificial Intelligence, University of Malta, Msida, Malta; ^2^Centre for Molecular Medicine and Biobanking, University of Malta, Msida, Malta

**Keywords:** virtual screening, machine learning, ultrafast shape recognition, ligand based virtual screening, ligand similarity, ElectroShape

## Abstract

Ultrafast Shape Recognition (USR), along with its derivatives, are Ligand-Based Virtual Screening (LBVS) methods that condense 3-dimensional information about molecular shape, as well as other properties, into a small set of numeric descriptors. These can be used to efficiently compute a measure of similarity between pairs of molecules using a simple inverse Manhattan Distance metric. In this study we explore the use of suitable Machine Learning techniques that can be trained using USR descriptors, so as to improve the similarity detection of potential new leads. We use molecules from the Directory for Useful Decoys-Enhanced to construct machine learning models based on three different algorithms: Gaussian Mixture Models (GMMs), Isolation Forests and Artificial Neural Networks (ANNs). We train models based on full molecule conformer models, as well as the Lowest Energy Conformations (LECs) only. We also investigate the performance of our models when trained on smaller datasets so as to model virtual screening scenarios when only a small number of actives are known *a priori*. Our results indicate significant performance gains over a state of the art USR-derived method, ElectroShape 5D, with GMMs obtaining a mean performance up to 430% better than that of ElectroShape 5D in terms of Enrichment Factor with a maximum improvement of up to 940%. Additionally, we demonstrate that our models are capable of maintaining their performance, in terms of enrichment factor, within 10% of the mean as the size of the training dataset is successively reduced. Furthermore, we also demonstrate that running times for retrospective screening using the machine learning models we selected are faster than standard USR, on average by a factor of 10, including the time required for training. Our results show that machine learning techniques can significantly improve the virtual screening performance and efficiency of the USR family of methods.

## Introduction

The discovery and development of a new drug is a time-consuming process that can take 14 years to complete successfully, incurring a cost of about 2.5 billion US dollars (DiMasi et al., 2016). Virtual Screening (VS) is a search approach that leverages electronic databases of chemical compounds and modern computing resources to streamline this process. The aim of this process is to computationally pre-screen molecules to find those that are most likely to exhibit affinity for binding to a given target protein. In this way, laboratory time and resources associated with High Throughput Screening (HTS) can be drastically reduced by preferentially testing only the compounds that are more likely to become successful leads (Leach and Gillet, 2007). Advances in processing power and high-capacity storage as well as development of Big-Data techniques has made this process of molecular screening feasible, resulting in significant savings of time and cost and significantly streamlining the drug discovery cycle (Leach and Gillet, 2007; Lavecchia and Giovanni, 2013).

Ligand-Based Virtual Screening (LBVS) is underpinned by the concept of similarity as defined in the Similarity Property Principle, which simply states that similar molecules tend to exhibit similar properties (Johnson and Maggiora, 1990). Many LBVS methods exist, but in essence they all require two steps. First, is the generation of a descriptor which represents a molecule. Second, is the search for a quantitative distance function which given two descriptors pertaining to different molecules computes the similarity between these. Descriptors for a library of molecules are compared to a query molecule's descriptor, which typically exhibits bioactivity. The result is a similarity ranking of all the molecules in the library. The top molecules from this list, *i.e.* the most similar to the bioactive one, are moved forward for physical testing.

There are many different types of LBVS methods such as fingerprints, pharmacophore modelling, Quantitative Structure-Activity Relationship modelling (QSAR), Ultrafast Shape Recognition (USR), *etc*. LBVS methods may use physicochemical properties, 2D topology, 3D molecular shape, and other dimensions such as electrostatics, lipophilicity, *etc*. in their descriptor generation stage. Some methods use a combination of these features (*e.g.* SHAFTS uses both pharmacophores and 3D structure information (Liu et al., 2011). In the case of LBVS methods that use shape information, these may be broadly divided into alignment and alignment-free methods. Alignment methods build a 3D model of the query and target molecules which are then superimposed. A common metric is to calculate volume overlap between the aligned (superpositioned) models. Alignment-free methods do not require an alignment for the descriptor comparison and are generally more efficient. For a review of shape-based similarity methods please refer to Finn and Morris (2013).

Ultrafast Shape Recognition (USR) is an alignment-free LBVS technique (Ballester and Richards, 2007a; Ballester and Richards, 2007b) that distils molecular shape into a rotation-invariant descriptor vector made up of 12 real numbers. These descriptors are then compared directly using a modified Manhattan Distance metric in order to obtain a measure of similarity.

The greatest advantage of this method is the exceedingly concise way in which the shape of a molecule is condensed into a small 12-element descriptor. The comparison of such small descriptors is fast to compute and efficient to store. This significant feature of USR made it orders of magnitude faster than any other shape-based similarity method that existed at the time (Ballester and Richards, 2007a).

This method was developed in 2007, however, extensions to this algorithm have since been proposed that extend the purely shape-based descriptors of USR with other physicochemical properties of the molecule, examples of which are ElectroShape 4D (Armstrong et al., 2010), ElectroShape 5D (Armstrong et al., 2011) and USRCAT (Schreyer and Blundell, 2012), which respectively add atomic partial charges, lipophilicity, and atomic types to pure USR descriptors, obtaisning better virtual screening scores than the original USR algorithm.

Even though extensive research has been carried out in the application of machine learning techniques to structure-based as well as ligand-based virtual screening, to the best of our knowledge there has not been a study systematically applying machine learning to USR and USR-based descriptors. The aim is to improve virtual screening performance with respect to the standard USR method.

In this study, we use the datasets provided in Directory of Useful Decoys-Enhanced (DUD-E) to train machine learning models based on Gaussian Mixture Models, Isolation Forests, and Artificial Neural Networks using USR and ElectroShape 5D descriptors in order to explore the performance improvement achievable by abandoning the standard USR similarity metric based on the inverse Manhattan Distance function in favour of a full machine learning approach.

GMMs and Isolation Forests were chosen because they are unsupervised, one-class learning methods that can be trained only on positive examples, in a sense, mimicking the standard USR method of using actives as search templates. GMMs and Isolation Forests take different approaches to this one-class learning problem. The former is a generative model, aiming to learn the probability distribution governing the training examples, whilst the latter is an outlier detection model, which rather than find clusters in the training data, detects outlying points. Further to these two algorithms, we chose to explore the use of ANNs in this study. This is a supervised method in wide use that gives excellent performance in a varied range of domains. We chose this algorithm because it enabled us to compare the performances of the two unsupervised methods with a supervised model. One-class learning methods are interesting in virtual screening since DUD-E contains real active molecules but only putative inactives (hence termed decoys).

Ballester et al. (2009) determined that using the LECs as active search templates provides a good performance-speed balance when evaluating compound databases using USR. We, therefore train alternative models using full active molecule conformers as training data as well as using only the active LECs in order to determine the performance differences between the two approaches.

Additionally, we also train similar models based on successively smaller fractions of the available training dataset so as to gauge the performance degradation of our models with respect to training dataset size. A good performance achieved even with a small number of active training examples is desirable because often, only a small number of actives are known *a priori* at the commencement of a prospective virtual screening exercise.

Through this study we demonstrate the potential of these techniques in significantly improving their retrospective screening performance. Our models obtain performance improvements over the state-ofthe-art ElectroShape 5D algorithm of a similar magnitude to those obtained by ElectroShape 5D itself over the original USR method, which were on the order of a maximum improvement of 738% and mean improvement of 253% for full conformers and a maximum of 755% and mean of 283% for LECs.

### Ultrafast Shape Recognition

The USR technique was ideated by Ballester and Richards (2007a; 2007b) wherein they proposed a novel nonsuperpositional shape-based virtual screening technique meant to preserve the virtual screening performance of superpositional algorithms while obtaining the speed benefits of non-superpositional methods.

Ballester et al. point out that the 3D shape of a molecule can be encoded by taking the Euclidean distance of each atom to a predetermined number of centroids located within the space occupied by the molecule. The number and position of the centroids can be arbitrary, however, while pointing out that their selection had not been validated to be the optimal one, the authors chose four well-defined centroids as follows:

1. The molecular centroid (*ctd*)
2. The closest atom to *ctd* (*cst*)
3. The furthest atom from *ctd* (*fct*)
4. The furthest atom to *fct* (*ftf*).

**Figure 1:**
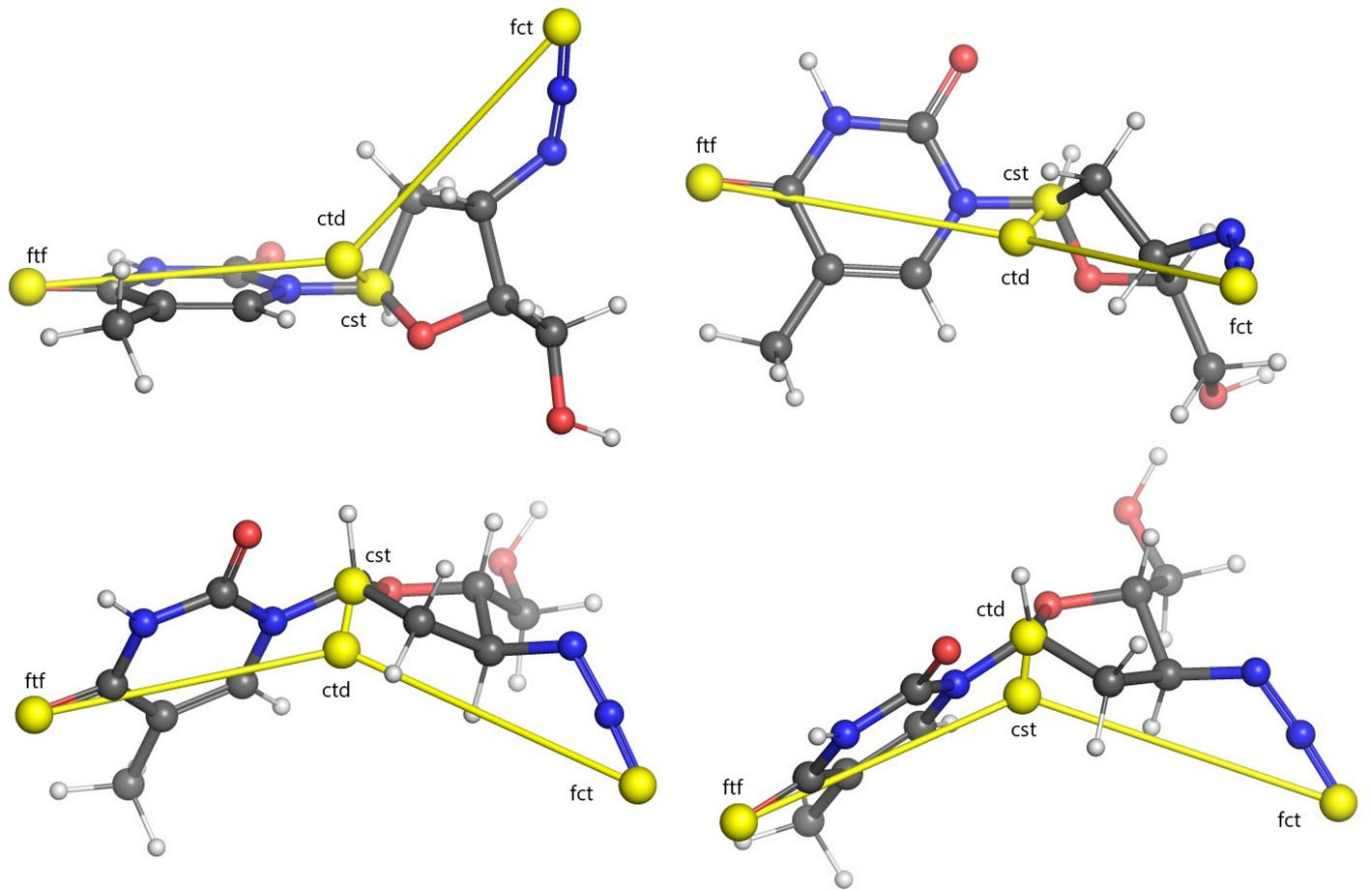
Illustration of USR centroids computed for a sample conformer of the Zidovoudine molecule. Centroids are indicated with yellow spheres. Lines between every centroid and the molecular centre are displayed for clarity. Four different rotations of the molecule are illustrated. Legend: ctd, molecular centroid; cst, closest atom to ctd; fct, furthest atom to ctd; ftf, furthest atom from fct.

Centroids computed for an example molecule are shown in **Figure 1**. Computing the Euclidean distances of all the atoms in the conformer to each of these four centroids yields four separate distance distributions of size proportional to the number of atoms making up the molecule.

As Ballester et al. indicate, however, there are several reasons these distributions are problematic to work with for the purposes of similarity searching. Most importantly, making use of these distributions as-is, it would not be possible to compare molecules having differing numbers of atoms because the distributions yielded by molecules of different sizes would also be of different sizes. In addition to this, distributions are normally represented as histograms, however this would still leave open the question of finding an optimal bin size given distributions of wildly differing sizes and characteristics generated from a database of molecules, not to mention the storage volume and processing power required for their processing.

They solve these problems by pointing out that a distribution is completely determined by its statistical moments (Hall, 1983), and condensing the four distributions into their respective first three moments, corresponding to the mean, the variance and the skewness of the distribution (Ballester and Richards, 2007a). This results in a vector of 12 decimal values making up a descriptor encapsulating shape information for a given conformer. The authors propose using this vector as a stand-in for the molecule's 3D structure in similarity comparisons. Ballester et al. (2009) modify this process by taking the square root and cube root of the second and third moments respectively, thus normalising them to a scale comparable to that of the first moment and resulting in better similarity matching performance.

The resulting descriptors could, in theory, be compared to each other using any similarity measure, however Ballester et al. chose to use a metric based on the Manhattan distance according to Equation 1.

(1) $$S_{qi}={\left(1+\frac{1}{12}\sum_{l=1}^{12}|M_{l}^{q}-M_{l}^{i}|\right)}^{-1}$$

where *S_qi_* gives a similarity value between the query conformer *q* and the conformer *i* being screened and ${\vec{M}}^{q}$ and ${\vec{M}}^{i}$ are the descriptor vectors for the query conformer and the conformer being screened, respectively. Here the sum is normalised by dividing it by the number of elements in the USR descriptor.

Ballester et al. (2009) formally evaluated the USR method comparing it to ESshape3D in terms of Enrichment Factor (EF) finding it to offer, on average, significantly better ranking performance. They furthermore pointed out that the ideal active conformers to use as search templates are those experimentally observed in their bound state *via* X-ray crystallography or MRI. When this is not available, however, they show that using the LECs is a good, but obviously not perfect, approximation. When using LECs they obtained retrospective virtual screening performance that is only slightly worse than the maximum possible enrichment.

As they point out, the method can be easily extended by incorporating into the descriptors other, nonspatial, atomic-centred information (Ballester et al., 2009). This was achieved by Armstrong et al. in a series of three papers—Armstrong et al. (2009); Armstrong et al. (2010) and Armstrong et al. (2011).

Armstrong's first effort at extending USR (Armstrong et al., 2009) was in the development of the Chiral Shape Recognition (CSR) method, aimed at overcoming the shortcoming of USR that enantiomers, i.e. molecules that are mirror images of each other, generate identical descriptors, however do not necessarily bind equally to a protein, causing false positives. Armstrong et al. modified the USR method to account for chirality in the descriptor calculation, thus eliminating this source of error and obtaining enrichment factor improvements of 121%, 113%, and 106% at 0.25%, 0.5%, and 1% EF respectively.

Subsequently, Armstrong et al. (2010) again modified CSR by incorporating atomic partial charges into its descriptors, resulting in a new method they called ElectroShape. They did this by adding an extra dimension to the descriptors, consisting of the partial charge pertaining to each atom scaled by a constant quantity *Q* so as to give them a magnitude comparable to the other spatial dimensions. This method resulted in a near doubling in performance over USR.

Armstrong et al. further extended their ElectroShape method in 2011 by adding lipophilicity in the form of ALogP to the ElectroShape descriptors in a similar manner as they had done for electrostatics, obtaining a further mean performance improvement of 110% over ElectroShape (Armstrong et al., 2011). This method shall hereafter by referred to as ElectroShape 5D.

Ultrafast Shape Recognition with CREDO Atom Types (USRCAT) is a further method that extends USR. Proposed by Schreyer and Blundell (2012), this method incorporates the atom types maintained in the CREDO Structural Interatomics Database (Schreyer and Blundell, 2009), these being hydrophobic, aromatic, hydrogen bond donor and hydrogen bond acceptor. It does this by computing separate distributions for each atom type, joining the resulting distribution moments into a single descriptor vector with 60 elements. USRCAT, on average, obtained a slightly higher average performance score than ElectroShape in retrospective screening on the DUD-E database with an *EF*_0_*. *_25%_ of 15.64 as opposed to 8.84 for USR and 14.48 for ElectroShape, however the exact performance depended on the target under consideration, with some targets scoring better than ElectroShape and others worse.

Other extensions to USR have also been proposed with a variety of modifications, ranging from the combination of USR descriptors with 2D fingerprints, incorporating atomic types and applying graph theory to the USR centroid concept (Cannon et al., 2008; Shave et al., 2015).

### Machine Learning Methods

Machine learning techniques have been applied extensively to virtual screening; both in Structure-Based Virtual Screening (SBVS) (Betzi et al., 2006; Ain et al., 2015; Wojcikowski et al., 2017) as well as LBVS where 2D fingerprints are naturally suited to be used as training data for machine learning algorithms (Stahura and Bajorath, 2004; Hert et al., 2006; Chen et al., 2007; Geppert et al., 2010; Kurczab et al., 2011; Lavecchia, 2015). This has, however, not been the case with USR, where to our knowledge, only Cannon et al. (2008) have applied machine learning to USR descriptors, and even then, in combination with 2D fingerprints.

In the work presented in this paper, we make an initial effort to fill this lacuna in current research related to USR, obtaining significant performance improvements over one of the highest performing USR-derived methods, ElectroShape 5D, by training several machine learning models on ElectroShape 5D descriptors.

LBVS can be considered as a ranking problem, where the objective is to sort molecules by similarity to one or more ligands that are used as search templates. We have chosen three machine learning algorithms to explore in this study, that are well suited to model this problem—GMMs, Isolation Forests, and ANNs.

A Gaussian Mixture Model (Reynolds, 2015) is a generative machine-learning model that models a distribution of data points using a combination of weighted Gaussian distributions. It can be considered to be a clustering algorithm similar to k-means (Hartigan and Wong, 1979); however, in a GMM, cluster membership of a data point is not absolute but instead is influenced probabilistically by several centroids. A GMM is described mathematically by Equation 2 below:

(2) $$f(x|\mu ,\text{Σ})=\sum_{k=1}^{M}c_{k}\frac{1}{\sqrt{2\pi \left|\Sigma_{k}\right|}}exp[\left(x-\mu_{k}{)}^{T}{\text{Σ}}_{k}^{-1}\left(x-\mu_{k}\right)\right]$$

where *M* is the number of Gaussians, also known as components, making up the GMM; *µ_k_* is the mean for component *k*; Σ*_k_* is the covariance matrix for component *k*, giving the co-variance between every pair of dimensions; and *c_k_* is the weight for component *k*. These number of components is a hyperparameter of the algorithm as is usually tuned through an iterative cross-validation process. The GMM is trained using the Expectation Maximization algorithm (Dempster et al., 1977).

GMMs have wide-ranging applications in machine learning. They have been used in speech recognition (Stuttle, 2003), audio speech classification (Siegler et al., 1997), for language and speaker identification (Reynolds, 1995; Reynolds and Rose, 1995), as well as in visual object tracking (Santosh et al., 2013) and image enhancement applications (Celik and Tjahjadi, 2011). They have also been used in virtual screening and, in particular, protein-ligand docking (Grant and Pickup, 1995; Grant et al., 1996; Jahn et al., 2010; Jahn et al., 2011).

Isolation forests (Liu et al., 2008) are a class of machine learning models known as ensemble models. Ensemble models make use of a collection of simpler models to improve their predictions over those that would have been obtained by any single one model. Isolation Forests are similar to the Random Forest algorithm (Ho, 1995) in that they create a number of Decision Trees (Breiman, 2017) based on the training data and averages the predictions from each decision tree to arrive at a final result. While Random Forests are a supervised algorithm used to perform classification tasks, Isolation Forests are unsupervised and are meant to be used to perform anomaly detection in a set of observations.

Contrary to other clustering algorithms which attempt to identify similar samples within the input dataset, Isolation Forests explicitly identify anomalies in the data. They do so by exploiting the fact that, averaged over a number of Decision Trees, the path length that will be needed to generate a prediction for an outlier will be, on average, significantly shorter than that required for an inlier observation.

The rationale for using Isolation Forests as an algorithm for ranking USR descriptors is by extension of the formal evaluation of the USR method by Ballester et al. (2009). Herein it was shown that upon clustering the conformers of the active molecules for a given protein, several cluster centroids emerge, corresponding to shapes matching the one or more binding modes presented by the target protein.

By definition, a large number of actives will fall on, or close to a given centroid, since most active molecules will have at least one conformer that matches a binding mode of the target protein. This means that, taking all the active conformers as a set, high-density zones should be apparent and centred around the cluster centroids. Non-binding conformers, on the other hand, will fall outside these high-density zones, making them into outliers or anomalies. Training an Isolation Forest using the descriptors for the active molecules and ranking these points by their anomaly score should yield results with good predictive power.

The third machine learning algorithm that we explored along the course of this study is the Artificial Neural Network (ANN). ANNs are models loosely inspired by the structure of the brain, being made up of several successive layers of nodes (neurons), each output of one layer of nodes feeding in to the inputs of the next.

The neural net is usually set up with an input layer having the same number of nodes as the number of features in the input data. The output of the input layer is then routed through one or more hidden layers and into an output layer which gives the result predicted by the network.

A single node *j* in layer *i* of a neural network consists of a vector of weights *W_i,j_* equal in length to the number of nodes in layer *i* − 1 and an activation function, which computes an output value for the neuron *a_i,j_* by taking into account the outputs of the previous layer *a_i_*_−_*_i_* and the corresponding weights *W_i_*.

There are a variety of activation functions that may be used in a neural network layer and it is possible to use different activation functions in different layers of a single network. Common ones include linear, sigmoid, and Rectified Linear Unit (RelU).

ANNs can be used for both classification as well as regression problems. For regression tasks, the output layer normally consists of one node with a linear activation function giving a real-valued output. For a classification network, the output layer is normally set up with one node for each class. The Softmax function, also called the Normalised Exponential Function, is applied to the outputs resulting a set of probabilities over the output classes.

In the context of molecule similarity ranking, regression networks are clearly the type of neural network that are the most suitable and the type of network used in this study. In our experiments, we used RelU activation for our hidden layer and linear activation on the output layer. The RelU activation is simple and is described by Equation 3 below:

(3) $$f\left(x\right)=\{\begin{array}{ll} 0, & \text{if }x\leq 0\\ x, & \text{if }x\geq 0 \end{array}$$

The linear activation function is also simple: *f*(*x*) = *x*.

Our intention in the selection of these three particular machine learning algorithms for our study was primarily to explore one-class learning models. Additionally, the “traditional” virtual screening process only involves using the known actives as “templates” against which to compare candidate molecules and not any decoys. Translating this into the machine-learning domain, this could be compared to one-class learning methods that, unlike supervised binary classifiers, do not make use of negative examples, but only positive ones. For these reason, we focussed most of our resources on exploring one-class learning algorithms, as we believed they would be better suited to the LBVS problem. However, we selected ANN as a general-purpose, widely-used supervised algorithm against which to compare the performance of the other one-class learning algorithms.

## Methods

Most of the previous literature involving USR has been evaluated on the Directory of Useful Decoys (DUD) database of compounds (Huang et al., 2006), however shortcomings have since been identified in DUD (Mysinger et al., 2012). Actives in the dataset were not diverse enough to ensure unbiased results from virtual screening algorithms. Decoy selection was also not optimal as significant imbalance existed between the net charges of actives and decoys with 42% of the actives having a net charge versus only 15% of the decoys. In 2012, Mysinger et al. released a new and updated database named DUD-E which tackled these shortcomings (Mysinger et al., 2012). DUD-E provides active and decoy datasets for 102 protein targets with an average active/decoy ratio of 1:50. To our knowledge, only the USRCAT method has been evaluated on DUD-E. We, therefore, made the choice of using the DUD-E the purposes of training and evaluating our models.

As previous work was evaluated on the DUD database, for ease of comparison, we selected the DUD38 subset of targets provided by DUD-E which consists of 38 of the 40 targets in DUD. The protein targets we considered together with the respective number of actives, decoys and resulting conformers are shown in **Table 1**. We have also provided the dataset sizes on disk for the 3D conformers that we generated from the SMILES representations of the molecule datasets as well as the sizes of the descriptors generated from said conformer data. These can be seen in **Table S2** in the **Supplementary Material**.

**Table 1:** The list of 38 protein targets that we considered in this study along with the number of active and decoy molecules that were available for each protein target, and the respective number of active and decoy conformers we generated. These targets correspond to the “Dud38” subset in DUD-E.

Target	Description	Active Mols.	Decoy Mols.	Active Confs.	Decoy Confs.	Confs./mol (Actives)	Confs./mol (Decoys)
ACE	Angiotensin-converting enzyme	282	16,900	31,947	1,266,730	113	74
ACES	Acetylcholinesterase	453	26,250	55,549	2,153,887	122	82
ADA	Adenosine deaminase	93	5,450	7,786	332,177	83	60
ALDR	Aldose reductase	159	9,000	4,797	375,355	30	41
AMPC	Beta-lactamase	48	2,850	1,351	99,431	28	34
ANDR	Androgen Receptor	269	14,350	12,068	543,761	44	37
CDK2	Cyclin-dependent kinase 2	474	27,850	21,273	1,371,687	44	49
COMT	Catechol O-methyltransferase	41	3,850	1,262	147,125	30	38
DYR	Dihydrofolate reductase	231	17,200	16,679	873,009	72	50
EGFR	Epidermal growth factor receptor erbB1	542	35,050	41,580	2,405,525	76	68
ESR1	Estrogen receptor alpha	383	20,685	21,024	1,212,349	54	58
FA10	Coagulation factor X	537	28,325	38,757	2,087,845	72	73
FGFR1	Fibroblast growth factor receptor 1	139	8,700	9,232	535,529	66	61
GCR	Glucocorticoid receptor	258	15,000	12,111	652,595	46	43
HIVPR	Human immunodeficiency virus type 1 protease	536	35,750	67,552	3,436,686	126	96
HIVRT	Human immunodeficiency virus type 1 reverse transcriptase	338	18,891	16,576	836,334	49	44
HMDH	HMG-CoA reductase	170	8,750	22,037	827,459	129	94
HS90A	Heat shock protein HSP 90-alpha	88	4,850	4,918	235,367	55	48
INHA	Enoyl-[acyl-carrier-protein] reductase	43	2,300	3,900	118,362	90	51
KITH	Thymidine kinase	57	2,850	3,168	150,295	55	52
MCR	Mineralocorticoid receptor	94	5,150	3,960	215,697	42	41
MK14	MAP kinase p38 alpha	578	35,850	34,310	2,096,198	59	58
NRAM	Neuraminidase	98	6,200	6,030	325,337	61	52
PARP1	Poly [ADP-ribose] polymerase-1	508	30,050	18,925	1,242,760	37	41
PDE5A	Phosphodiesterase 5A	398	27,550	32,657	1,876,746	82	68
PGH1	Cyclooxygenase-1	195	10,800	8,123	410,263	41	37
PGH2	Cyclooxygenase-2	435	23,150	19,598	960,837	45	41
PNPH	Purine nucleoside phosphorylase	103	6,950	3,277	284,801	31	40
PPARG	Peroxisome proliferator-activated receptor gamma	484	25,300	71,166	2,527,881	147	99
PRGR	Progesterone receptor	293	15,650	13,041	578,492	44	36
PUR2	GAR transformylase	50	2,700	7,931	195,987	158	72
PYGM	Muscle glycogen phosphorylase	77	3,950	3,300	212,652	42	53
RXRA	Retinoid X receptor alpha	131	6,950	8,008	316,919	61	45
SAHH	Adenosylhomocysteinase	63	3,450	1,883	118,691	29	34
SRC	Tyrosine-protein kinase SRC	524	34,500	39,561	2,313,655	75	67
THRB	Thrombin	461	27,004	57,028	2,131,048	123	78
TRY1	Trypsin I	449	25,980	47,961	1,933,063	106	74
VGFR2	Vascular endothelial growth factor receptor 2	409	24,950	25,349	1,518,622	61	60

As with many virtual screening methods that depend on molecular 3D shape, a sufficient number of conformers have to be generated to adequately sample the molecules' conformational space in order to produce effective results in USR. We generated conformers from the Simplified Molecular Input Line Entry Specification (SMILES) strings provided in DUD-E using the RDKit open-source cheminformatics library (Landrum and Others, 2013) following the protocol devised by Ebejer et al. (2012).

Conformer generation is performed using open-source code by Steven Kearnes^1^ which follows the protocol laid out by (Ebejer et al., 2012). We modified this code in two ways:

- Use of ETKDG. We modified the code to use Experimental Torsion Knowledge Distance Geometry (ETKDG) as the conformer generation algorithm (Riniker and Landrum, 2015). ETKDG is a stochastic conformer generation method which builds upon the existing Distance Geometry (DG) algorithm (Blaney and Dixon, 1994) by using experimental knowledge about preferential torsional-angles. The major advantage in using ETKDG as opposed to DG is that the output of DG is not optimal and the resulting conformers may be in a distorted state (e.g., aromatic rings which are not planar). In order to remedy this, a second energy minimisation step is usually performed on these conformers in which inter-atomic force-field calculations are used to relax the molecule into a stable, energy-minimized state. This computationally expensive step is avoided by Experimental-Torsion Knowledge Distance Geometry (ETKDG) as the embedded knowledge in the algorithm produces conformers that are already energy minimized.
- Maximum energy cutoff. We removed all conformers which had a total energy higher than that of the LEC by 5 kcal/mol or more. This ensures that conformers with high energy (typically unsound structures) are discarded.

Prior to conformer generation, we validated and standardized the molecules using the MolVS tool^2^. This tool has been now integrated into RDKit.

Once we had generated a sufficient number of conformers for the compounds pertaining to our chosen protein targets, we calculated USR descriptors as well as descriptors for CSR, ElectroShape, and ElectroShape 5D for all the generated conformers. Note, however, that for reasons of time and resource availability, we chose to perform our machine learning experiments exclusively on the descriptors for USR and those for ElectroShape 5D. ElectroShape 5D was chosen because it is the highest performing USR-like method among those we evaluated.

The processes of conformer and descriptor generation resulted in excess of 300 GB of data. In order to generate and process this in a feasible amount of time, we used a Python 3.6/Spark 2.3.0 cluster on Amazon Web Services consisting of 3 compute-optimised c5.2xlarge instances having 8 cores and 16 GB of memory each. Cheminformatics analysis was performed using RDKit (version 2018.09.1). We also used the machine learning algorithms supplied with version 0.20.2 of the Scikit-learn library as well as Keras v.2.2.4/Tensorflow v.1.14.

### Experiments

The first experiments that we conducted were retrospective virtual screening using both USR and ElectroShape 5D over all the DUD38 protein targets in DUD-E. This gave us a baseline performance level against which to compare the results of the machine learning experiments.

For both USR as well as ElectroShape 5D, two versions of the experiments were performed.

The first used the full molecule conformer models of the actives as search templates for the similarity matching, comparing each conformer of each unknown molecule to each conformer of the template, taking the maximum similarity as the similarity score between the two molecules.

The second used only the LEC for each active as the search templates rather than all the active conformers in order to replicate the results of Ballester et al. (2009).

Having obtained baseline performance measures for the standard Manhattan distance-based USR and ElectroShape 5D screening processes, we proceeded to train the three types of machine learning models described previously.

Our training protocol was similar for all three algorithms and is described as follows:

1. Partition the training set into test set *T* (20%) and training set *L* (80%).
2. Partition *L* set into 5 folds, *L*_1_*…L*_5_.
3. For every choice of hyperparameter (grid search), perform 5-fold cross validation on *L*, i.e. perform training and testing over *j* = 1*…*5 iterations, each time taking *L_j__=__x_* as a test set and the 4 folds *L_j_*_≠_ *_x_* together as training set.
4. Select highest scoring grid search hyperparameter value combination averaged over the 5 iterations.
5. Train model using highest performing hyperparameter combination using *L* as the training set and *T* as the test set to evaluate final model. This ensures that the final test set is completely disjoint from the training data and avoids bias in the final results.

This process was repeated for every protein target at successively smaller portions of the entire dataset available in DUD-E equivalent to 100%, 80%, 60%, 50%, 30%, 10%, 5%, and 10 molecules, selected at random. All this is furthermore repeated for models trained using full molecule conformer models and for LECs models, running the training/testing cycle for a total of 16 times per protein target.

### Evaluation

For every model trained, we evaluated the performance using two criteria—the Receiver Operator Characteristic (ROC) Area Under Curve (AUC) and the EF. EF is a measure used specifically in retrospective virtual screening studies. EF at a given percentage of a dataset is defined as the ratio of the fraction of actives correctly found within the first x% of the ranked dataset to the fraction of actives that would be found by chance. This is defined formally in Equation 4.

$$EF_{x\%}=\frac{a_{x\%}/c_{x\%}}{a_{100\%}/c_{100\%}}$$

where *EF_x_*_%_ is the enrichment factor at x%, *a_x_*_%_ is the number of actives found in the top x% of the sorted dataset and *c_x_*_%_ is the total number of compounds in x% of the dataset. This measure, however, depends on the ratio of decoys to actives that are present in the dataset, and therefore is problematic to use when comparing results across different studies. For this reason, we also evaluate our models based on the ROC AUC.

The disadvantage to using ROC AUC performance metric, in the context of retrospective virtual screening, is that they give a picture of the performance of the method across the entire dataset, however in virtual screening only the top-ranked molecules are of interest. This is because in a prospective screening scenario, it is not possible to physically test all the compounds in the dataset and the available resources for testing in the laboratory would be invested only on the best-ranked compounds.

Unlike the EF, the ROC AUC does not depend on the structure of the dataset, making it more suitable and robust when used for comparison across studies using different benchmark datasets.

## Results

**Figure 2:**
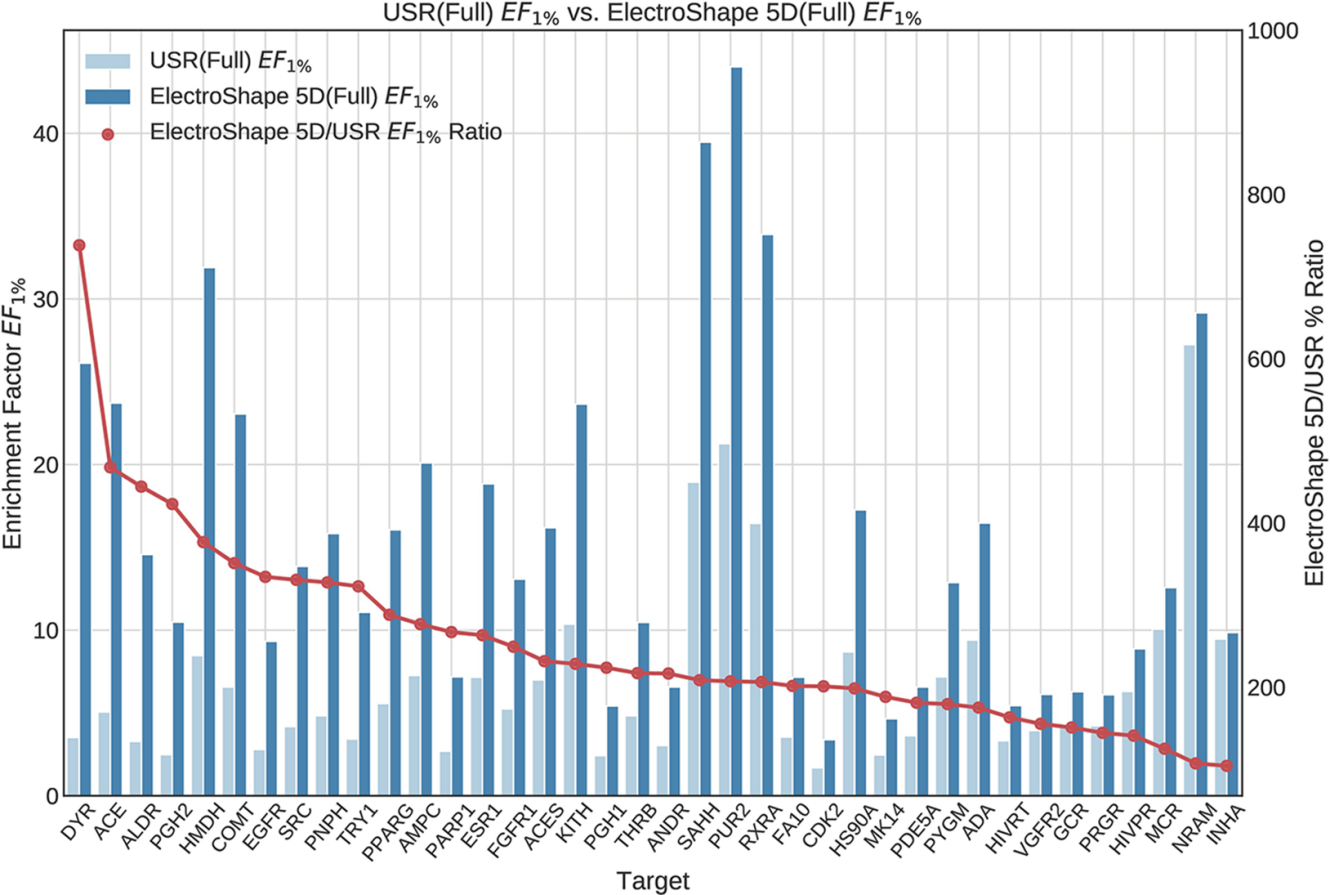
Comparison of Enrichment Factor at 1% (*EF*_1%_) obtained by USR with that obtained by ElectroShape 5D using full conformer models. Also plotted is the percentage ratio of the Enrichment Factor score of ElectroShape 5D compared to Ultrafast Shape Recognition (USR). Mean ratio = 253% ± 122%, max = 738%, min = 104%.

**Figure 3:**
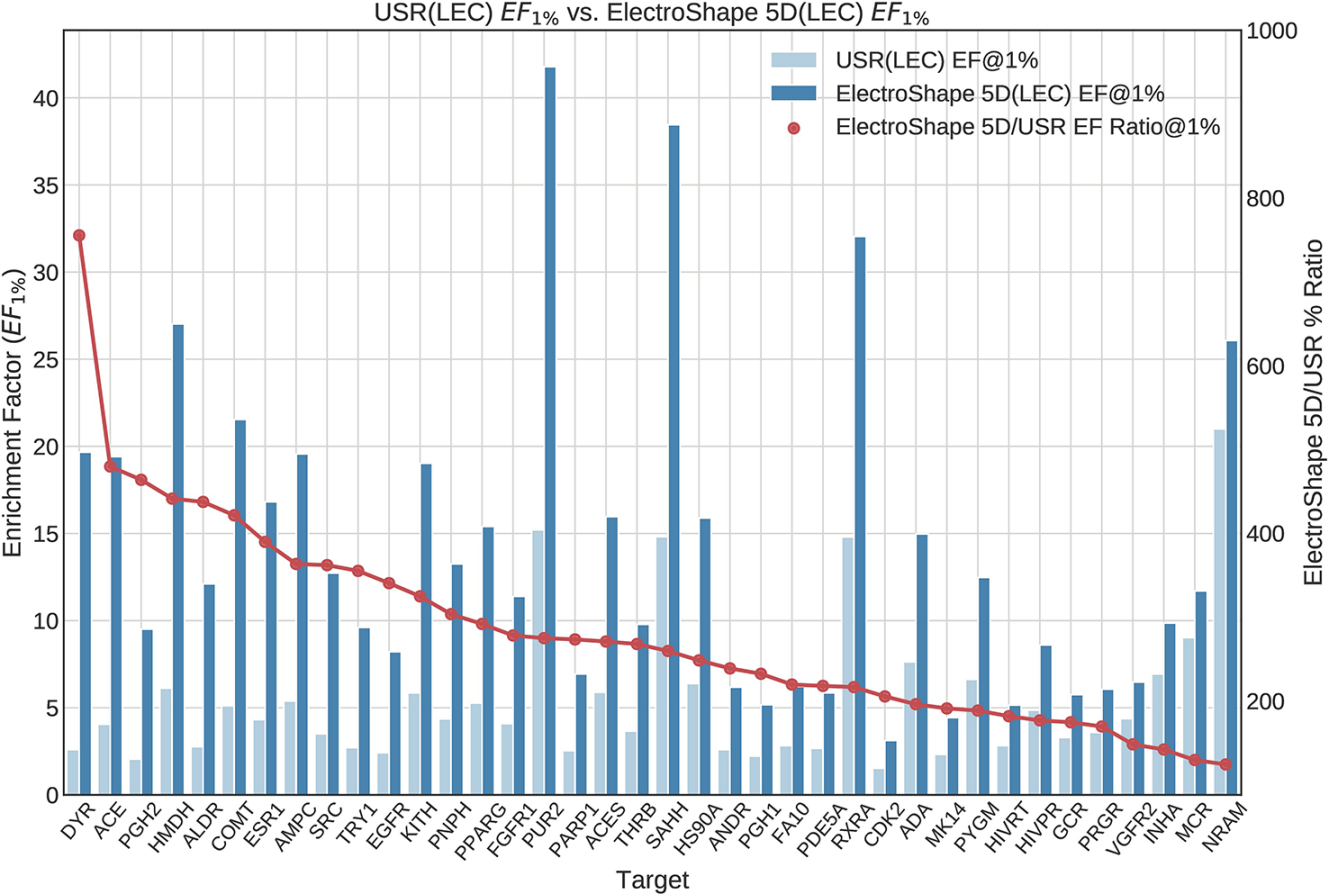
Comparison of Enrichment Factor at 1% obtained by USR with that obtained by ElectroShape 5D using Lowest Energy Conformers. Also plotted is the percentage ratio of the Enrichment Factor score of ElectroShape 5D compared to Ultrafast Shape Recognition (USR). Mean ratio = 283% ± 125%, max = 755%, min = 124%.

The first stage in our experiments was to implement and evaluate the standard USR and ElectroShape 5D methods. Evaluation of our results with those of Ballester et al. (2009) and Armstrong et al. (2011) show them to be comparable albeit with differences, since they are evaluated on different datasets with a different decoy selection. Our results are shown in **Figures 2** and **3**. As can be seen, ElectroShape 5D obtains better performance than standard USR in all the protein targets being considered. The corresponding ROC AUC measures can be seen in the **Supplementary Material**.

**Figure 4:**
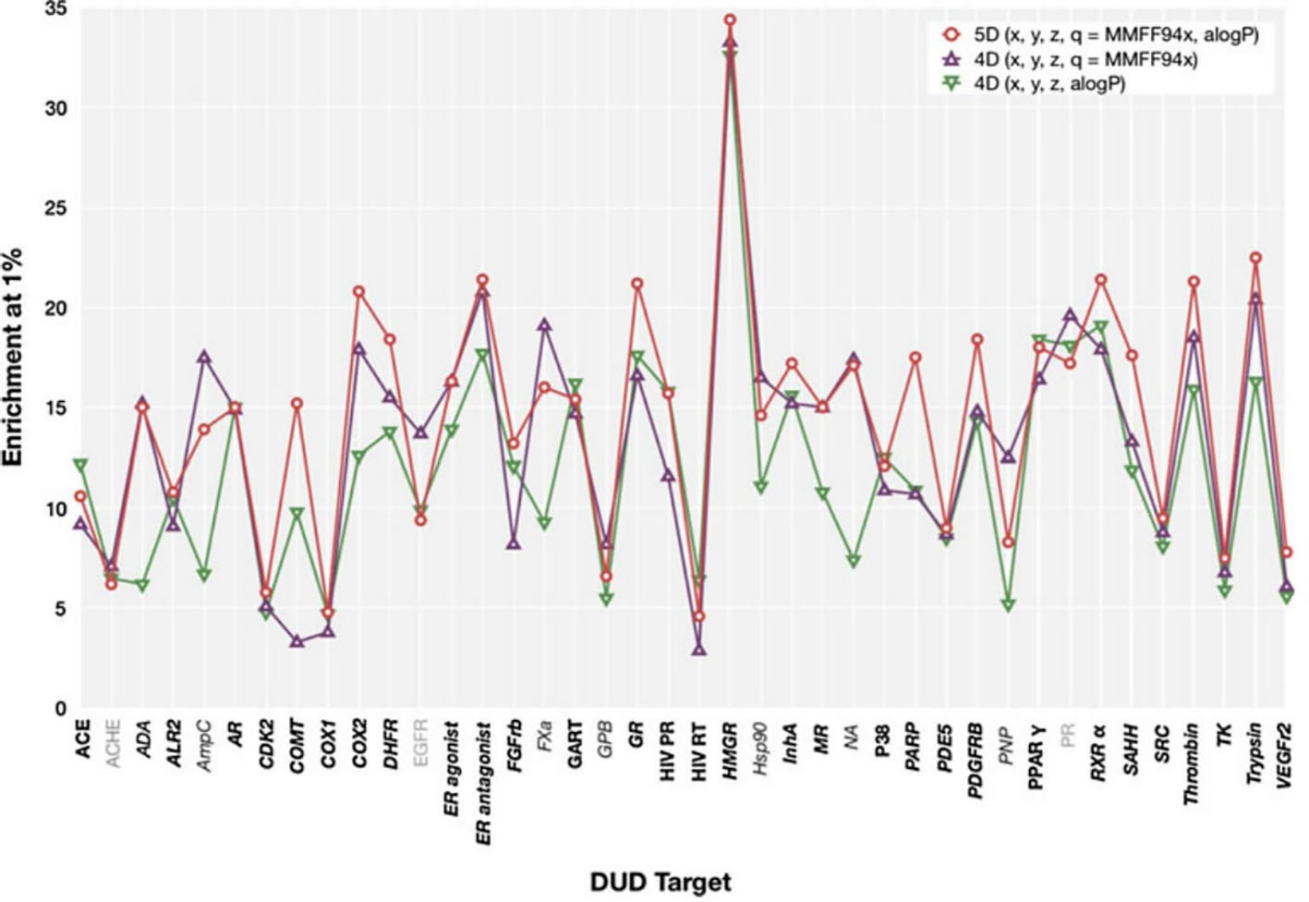
ElectroShape 5D *EF*_1%_ calculated on the DUD dataset as reported in Armstrong et al. (2011). Legend: 5D(x,y,z,q = MMFF94x,aLogP)—ElectroShape 5D with partial charge and lipophilicity as the 4th and 5th dimensions, 4D(x,y,z,q = MMFF94x)—ElectroShape 4D using partial charge as the 4th dimension, 4D(x,y,z,q = aLogP)—ElectroShape 4D using lipophilicity as the 4th dimension. Reproduced from Armstrong et al., 2011.

We observed that, in general, our results show a similar trend to those presented by Armstrong et al. (2011) (reproduced in **Figure 4**), i.e., most targets that show a high enrichment in our results also show a high enrichment in Armstrong's results and vice versa, but there are differences. The Pearson productmoment correlation coefficient for the two sets of data is 0.35, indicating a mild positive correlation. Given the differences in decoy selection in DUD-E in comparison with DUD (Mysinger et al., 2012), it is not surprising that our results differ from those obtained by Armstrong. This relatively low, albeit positive, correlation coefficient, indicates that differences in dataset selection can have a significant impact on virtual screening results.

**Figure 5:**
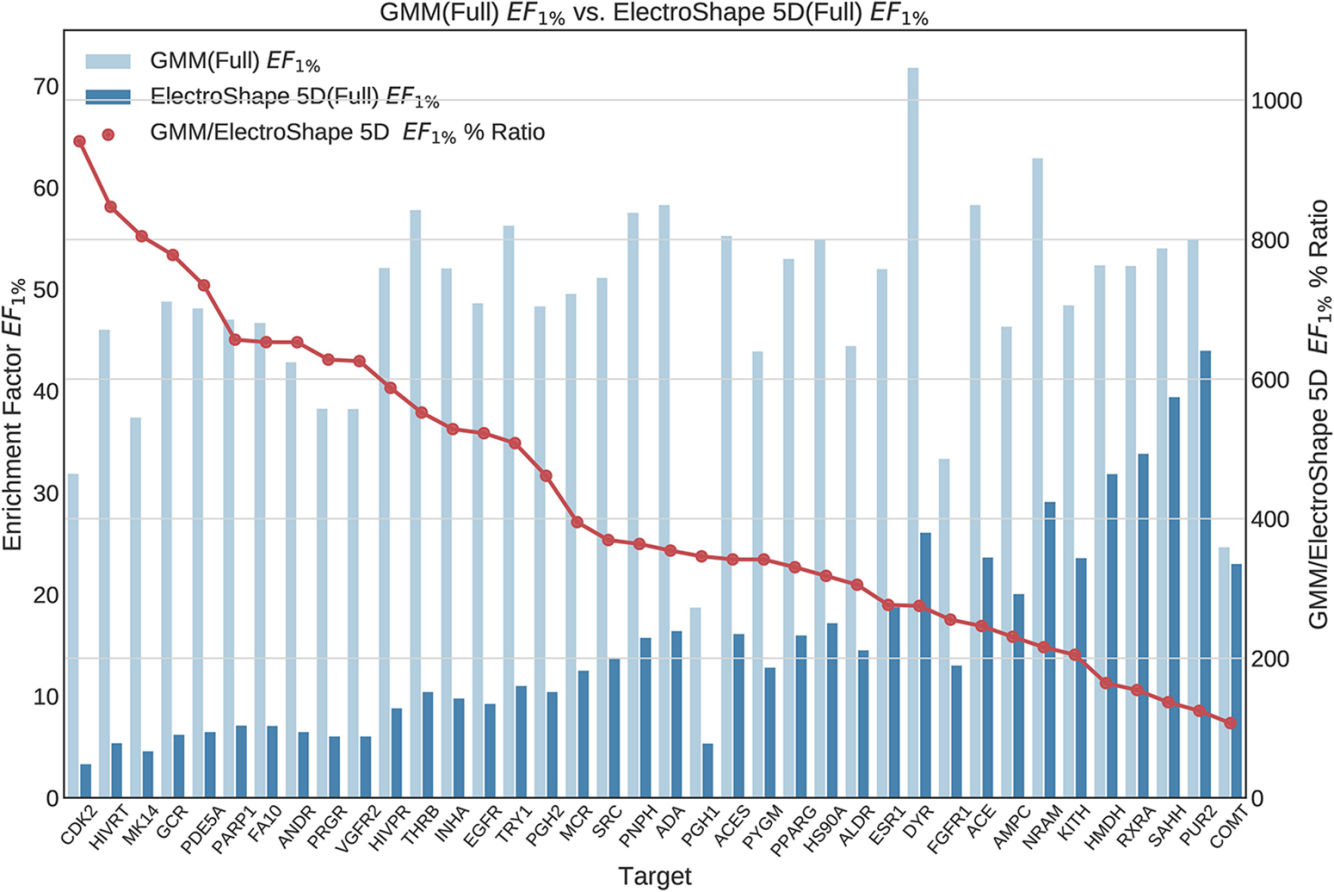
Comparison of Enrichment Factor at 1% obtained by Gaussian Mixture Models with that obtained by ElectroShape 5D using full conformer model. Also plotted is the percentage ratio of the Enrichment Factor score of Gaussian Mixture Model (GMM) compared to ElectroShape 5D. Mean ratio = 430% ± 223%, max = 941%, min = 107%.

**Figure 6:**
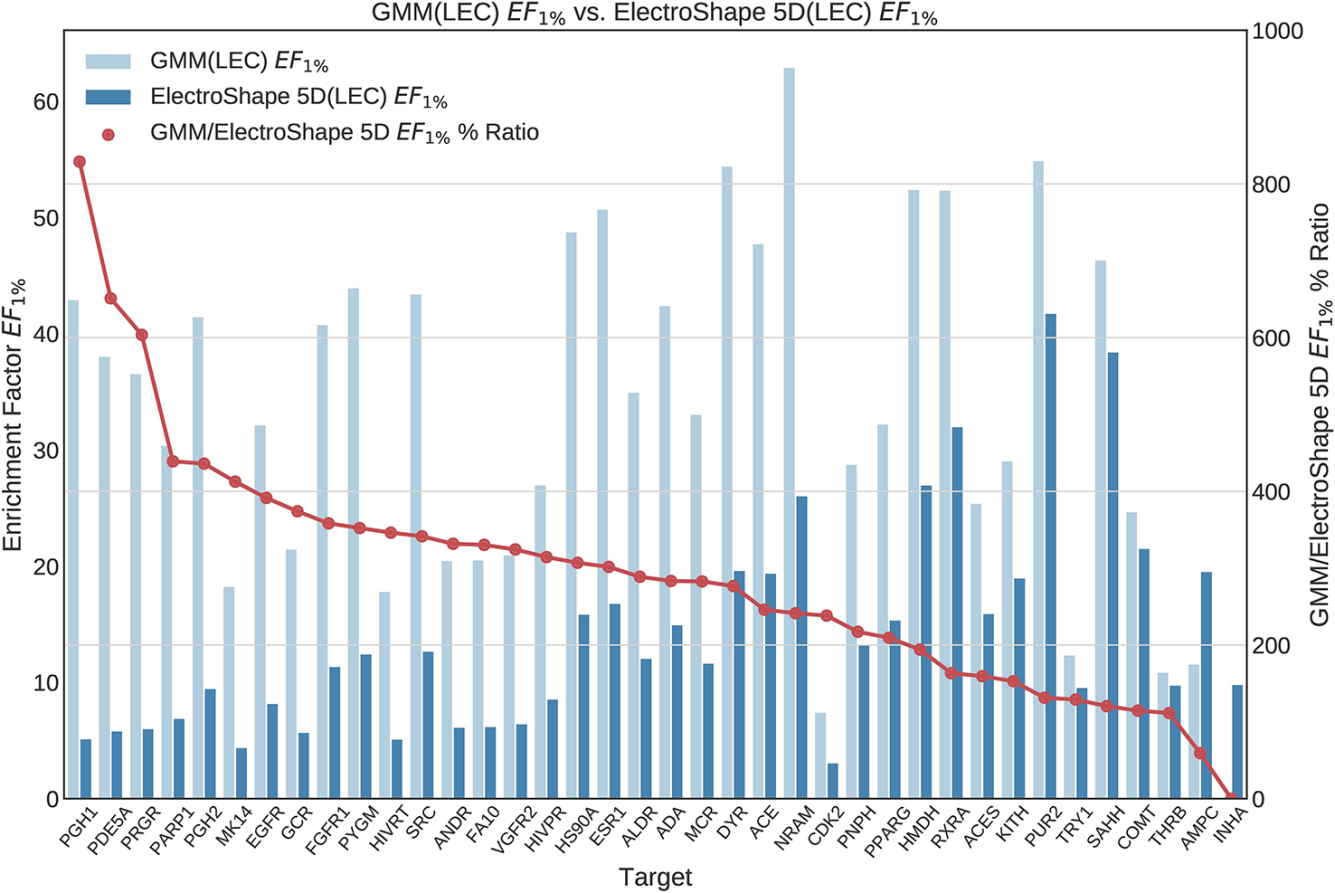
Comparison of Enrichment Factor at 1% obtained by Gaussian Mixture Model with that obtained by ElectroShape 5D using Lowest Energy Conformers. Also plotted is the percentage ratio of the Enrichment Factor score of Gaussian Mixture Model (GMM) compared to ElectroShape 5D. Mean ratio = 291% ± 162%, max = 829%, min = 0%.

**Figure 7:**
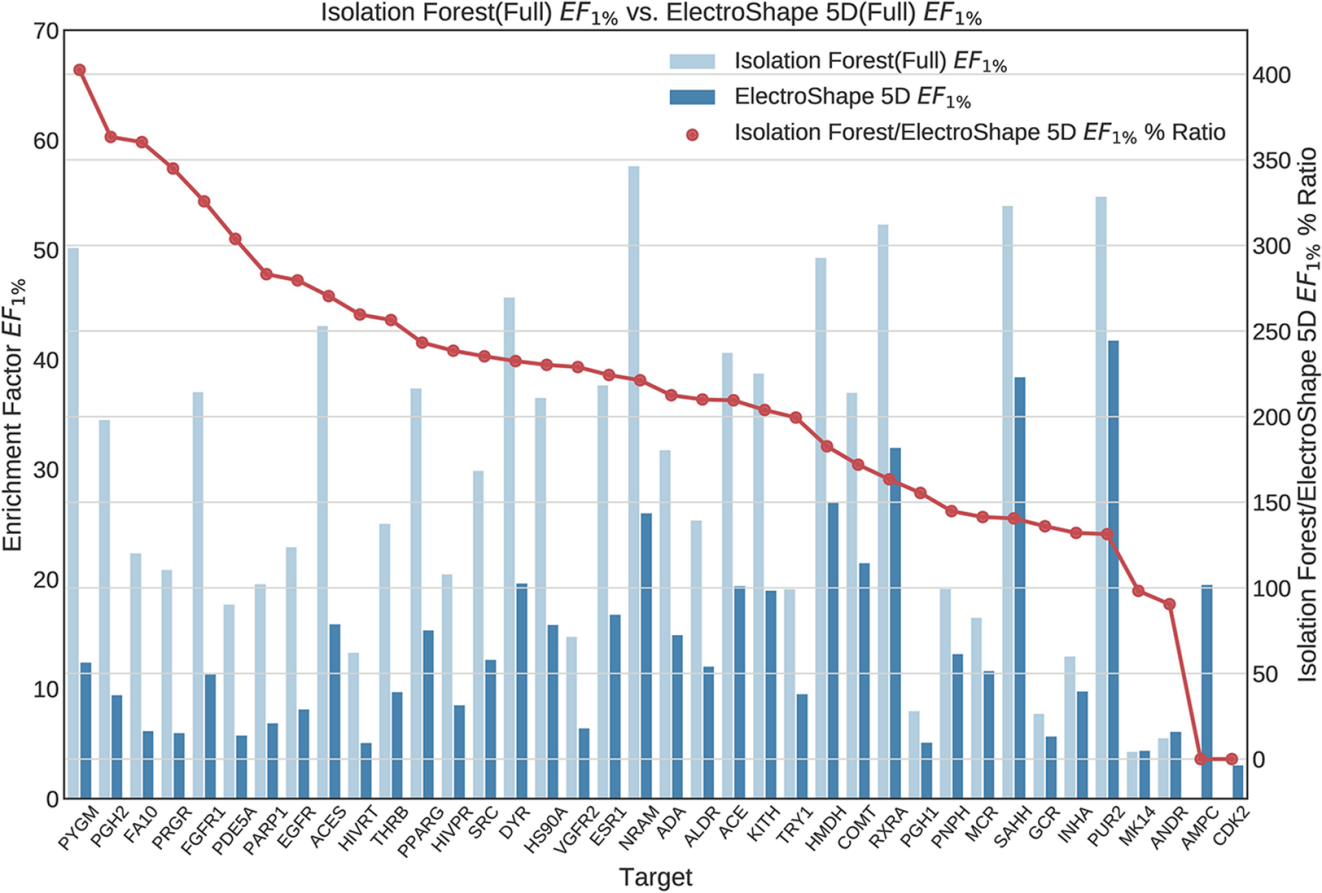
Comparison of Enrichment Factor at 1% obtained by Isolation Forest with that obtained by ElectroShape 5D using full conformer model. Also plotted is the percentage ratio of the Enrichment Factor score of Isolation Forest compared to ElectroShape 5D. Mean ratio = 211% ± 90%, max = 941%, min = 107%.

**Figure 8:**
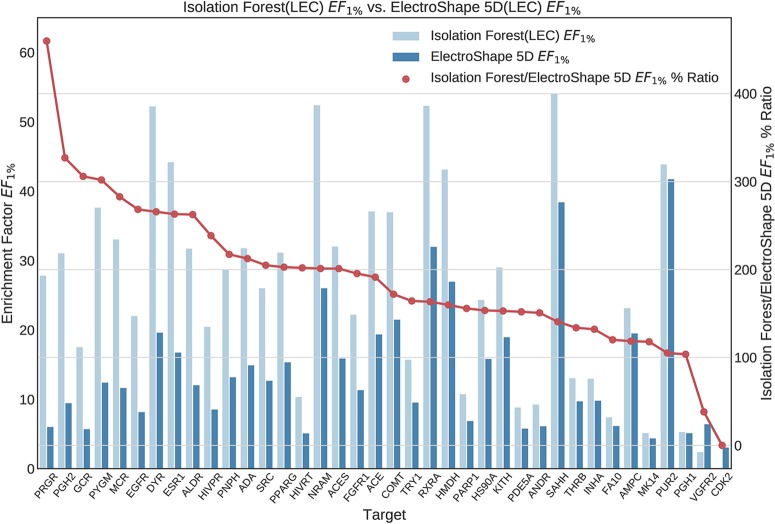
Comparison of Enrichment Factor at 1% obtained by Isolation Forest with that obtained by ElectroShape 5D using Lowest Energy Conformers. Also plotted is the percentage ratio of the Enrichment Factor score of Isolation Forest compared to ElectroShape 5D. Mean ratio = 190% ± 84%, max = 460%, min = 0%.

**Figure 9:**
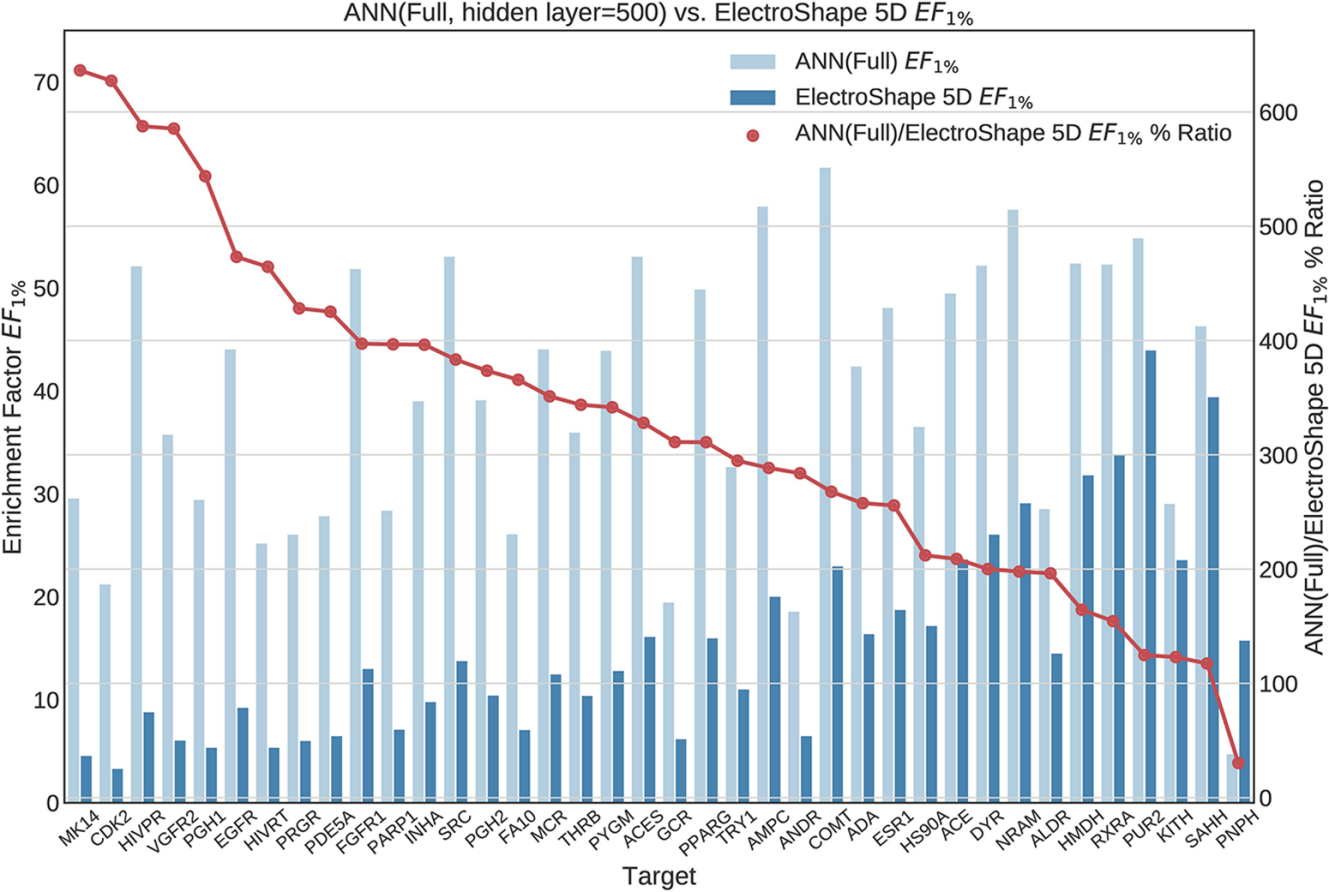
Comparison of Enrichment Factor at 1% obtained by Artifical Neural Networks with 500-node hidden layer with that obtained by ElectroShape 5D using full conformer models. Also plotted is the percentage ratio of the Enrichment Factor score of Artificial Neural Network (ANN) compared to ElectroShape 5D. Mean ratio = 328% ± 149%, max = 636%, min = 30%.

**Figure 10:**
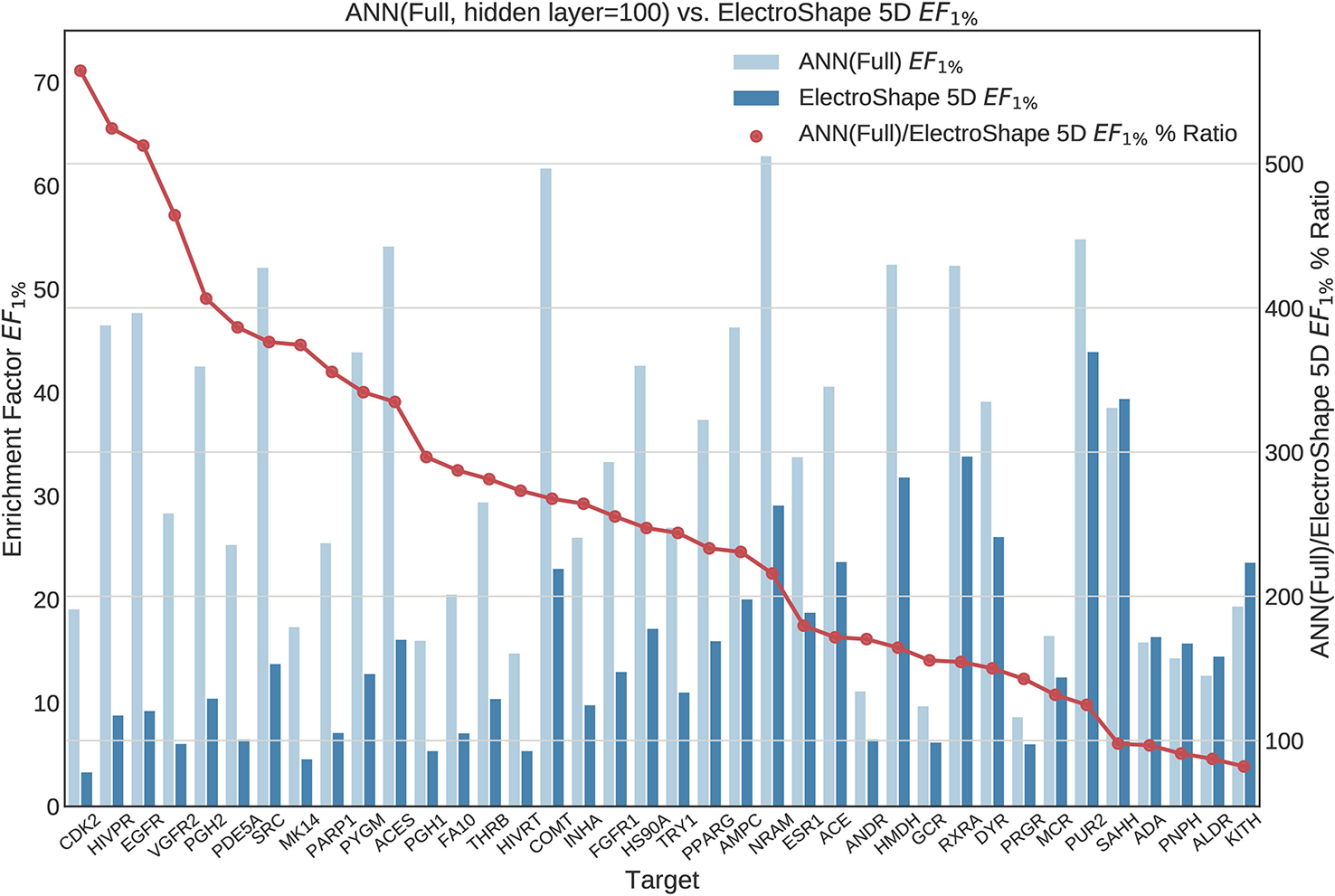
Comparison of Enrichment Factor at 1% obtained by Artificial Neural Networks with 100-node hidden layer with that obtained by ElectroShape 5D using full conformer models. Also plotted is the percentage ratio of the Enrichment Factor score of Artificial Neural Network (ANN) compared to ElectroShape 5D. Mean ratio = 256% ± 129%, max = 565%, min = 82%.

**Figure 11:**
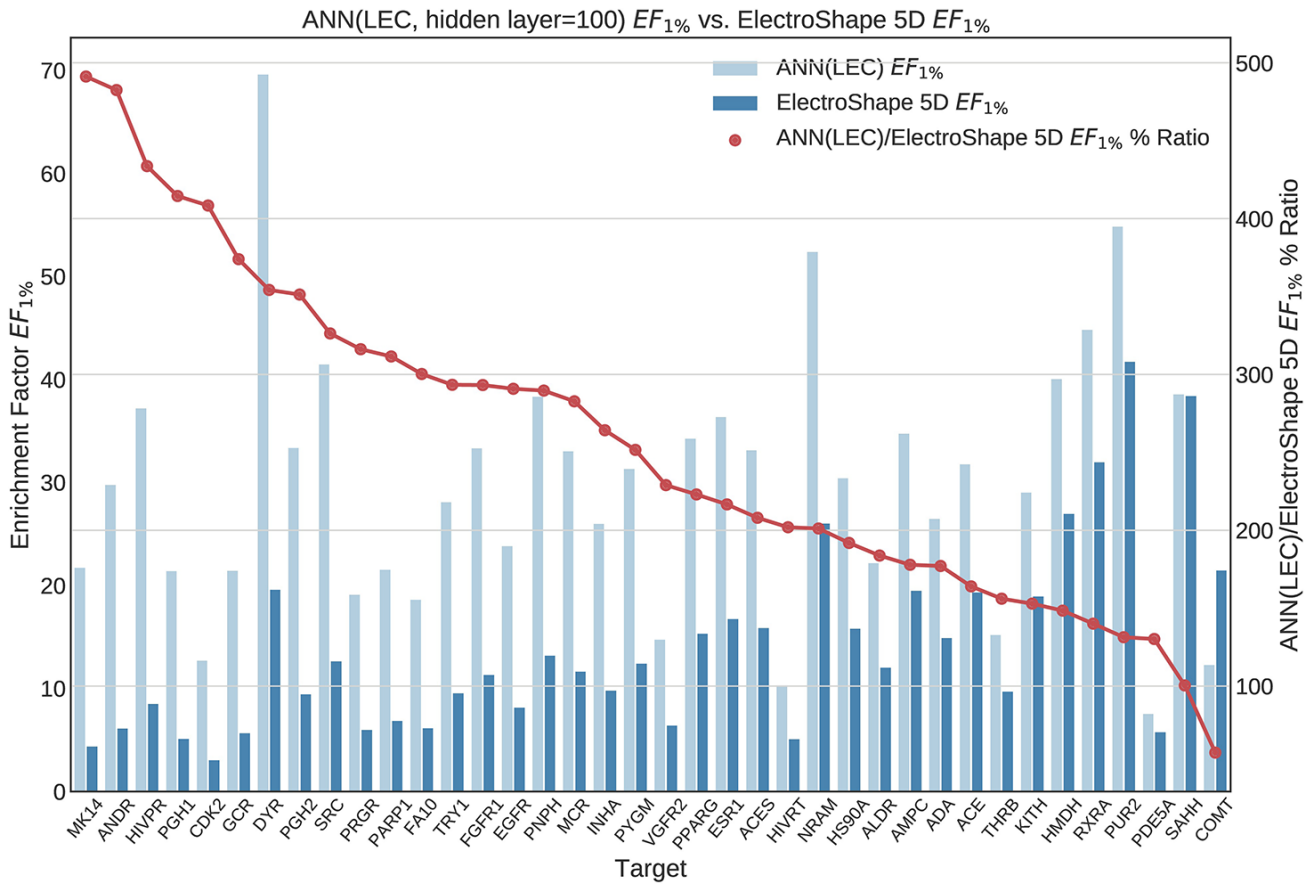
Comparison of Enrichment Factor at 1% obtained by Artifical Neural Networks with 100-node hidden layer with that obtained by ElectroShape 5D using Lowest Energy Conformers. Also plotted is the percentage ratio of the Enrichment Factor score of Artificial Neural Network (ANN) compared to ElectroShape 5D. Mean ratio = 256% ± 107%, max = 491%, min = 57%.

Once we generated results for our baseline methods, we trained and evaluated our machine learning models as described in the section *Experiments*. The results obtained from our machine learning experiments are visualised as follows. For each machine learning model, we have graphed the *EF*_1%_ as well as the ROC AUC achieved by the model along with the corresponding evaluation result achieved by ElectroShape 5D. Along with these we graph the improvement ratio between the performance of the model and the performance of ElectroShape 5D so as to indicate immediately the advantage in performance afforded by the use of the machine learning method over ElectroShape 5D for every protein target. We do this for models trained on full conformer models as well as for those trained on LECs. Due to space constraints, we only present the *EF*_1%_ results. These can be seen in **Figures 5**–**11**. A complete set of visualisations is made available in the **Supplementary Material** (**Figures S1**–**S15**).

All the results obtained by the machine learning models we trained are presented in tabular form in **Table 2**.

**Table 2:** Summary of machine learning results expressed as percentage ratios over ElectroShape 5D. A value of 100% indicates that the same performance as ElectroShape 5D was obtained.

LEC	GMM	Isolation Forest	ANN	ANN
			(Hidden layer = 100)	(Hidden layer = 500)
Mean *EF*_1%_(± std)	291%(± 162%)	191%(± 084%)	256%(± 107%)	333%(± 130%)
Max *EF*_1%_	829%	450%	491%	618%
Min *EF*_1%_	0%	0%	57%	121%
Mean AUC(± std)	133%(± 17%)	126%(± 15%)	139%(± 17%)	144%(± 21%)
Max AUC	171%	155%	175%	179%
Min AUC	104%	99%	104%	105%
Full Conformers				
Mean *EF*_1%_(± std)	430%(± 223%)	211%(± 90%)	256%(± 129%)	328%(± 149%)
Max *EF*_1%_	941%	403%	565%	636%
Min *EF*_1%_	107%	0%	82%	30%
Mean AUC(± std)	137%(± 19%)	124%(± 14%)	136%(± 20%)	143%(± 20%)
Max AUC	173%	153%	173%	177%
Min AUC	105%	99%	103%	104%

Note that when training the ANNs, we expected to see a performance drop in the LEC model with respect to the full conformer-trained model, as for the other models, however, training both using a hidden layer size of 100 nodes, this did not materialise and the performance obtained for the LEC-trained model, in terms of mean *EF*_1%_ improvement ratio over ElectroShape 5D, was virtually the same for the same hidden layer size (255% ± 106% vs. 256% ± 129%). Upon increasing the hidden layer size to 500 nodes, this situation did not change (333% ± 128% vs. 327% ± 148%). It is also interesting that the ANN performance did not surpass that of the full-conformer GMM. Based on these results, the ANN model does not perform as well as GMMs.

It is also important to note that the imbalance in the training datasets, i.e., the ∼1:50 active/decoy ratio, can cause some supervised machine learning models such as ANNs to give misleading test results by adapting their response to the distribution of labels in the training data rather than to the structure of the data itself. We verified the effect of the DUD-E unbalanced datasets on our ANN models by training alternative models using oversampling of the active conformers to balance the active/decoy ratio. Through these experiments we saw that the results obtained by balancing the datasets were comparable to those obtained from the unbalanced ones (mean unbalanced ROC AUC = 0.937 ± 0.037 vs. balanced ROC AUC = 0.955 ± 0.33, mean unbalanced *EF*_1%__=_ 38.2 ± 11.7 vs. mean balanced *EF*_1%__=_ 37.3 ± 14.7). Balancing the datasets in this way, however results in almost twice the training data for each model that is trained, and therefore a correspondingly longer training time. Given the marginal differences in results obtained through these experiments, therefore, we stuck to using the original unbalanced data to train our ANNs. Note that dataset balance is not an issue with either GMMs or Isolation Forests since decoys are not used when training these models.

### Varying the Size of the Training Dataset

We have repeated our experiments for every machine learning algorithm multiple times using successively smaller portions of the available dataset so as to explore the manner in which the performance given by each model degrades with dataset size and to understand how the performance of machine learning models degrades with a reduced dataset.

**Figures 12** and **13** contain plots illustrating the performance variation with number of known actives of our GMM models, the best performing models in our tests. The complete set of figures illustrating the performance change with dataset size for all our trained models can be found in the **Supplementary Material** (**Figures S1**–**S15**).

**Figure 12:**
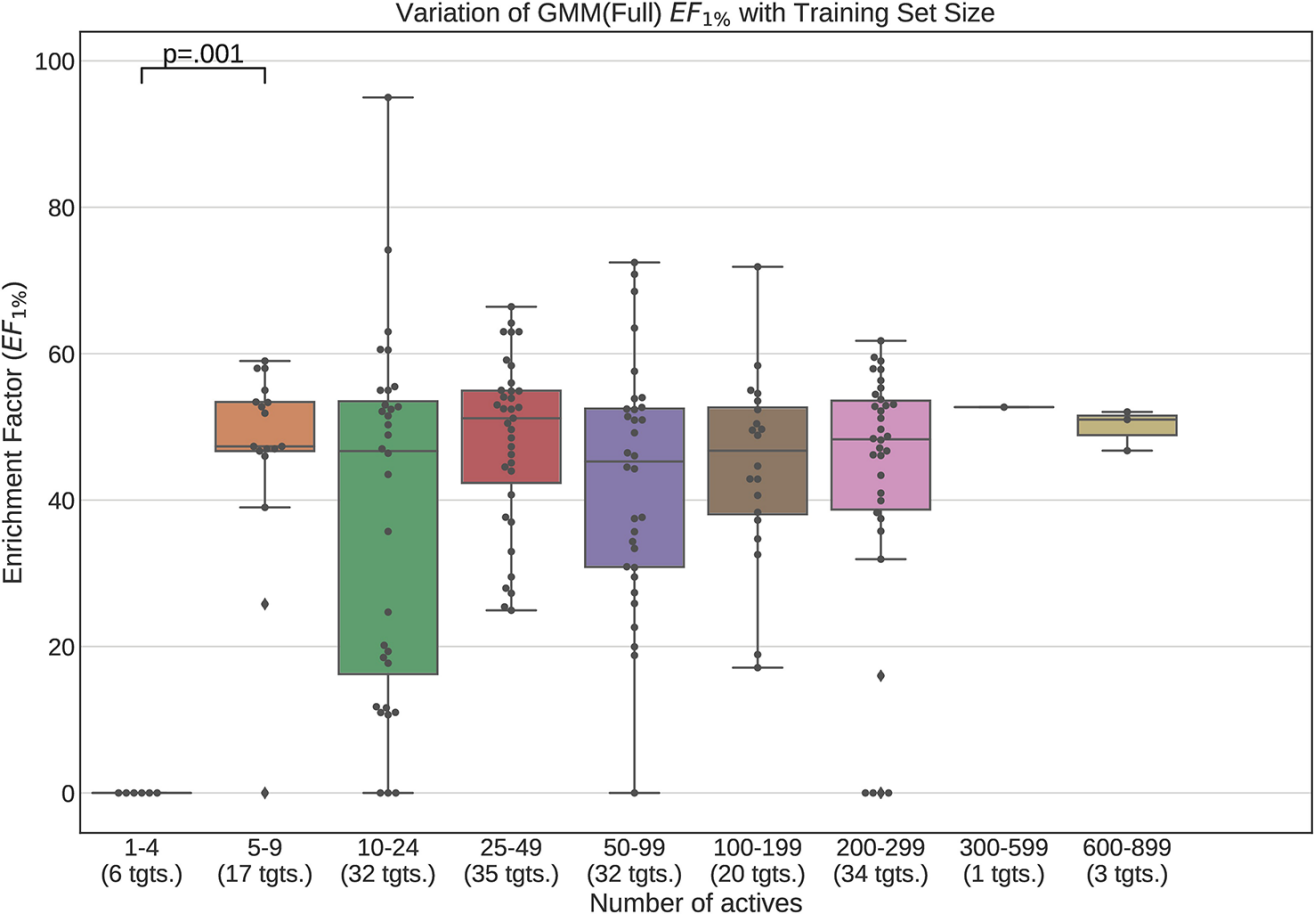
Performance variation of full-conformer model Gaussian Mixture Models with number of actives. Scatter plot indicates one point per template within the given range. The number of templates captured within the range is indicated in the axis labels. Note that multiple points belonging to the same target could fall within a single range due to the binning thresholds used.

**Figure 13:**
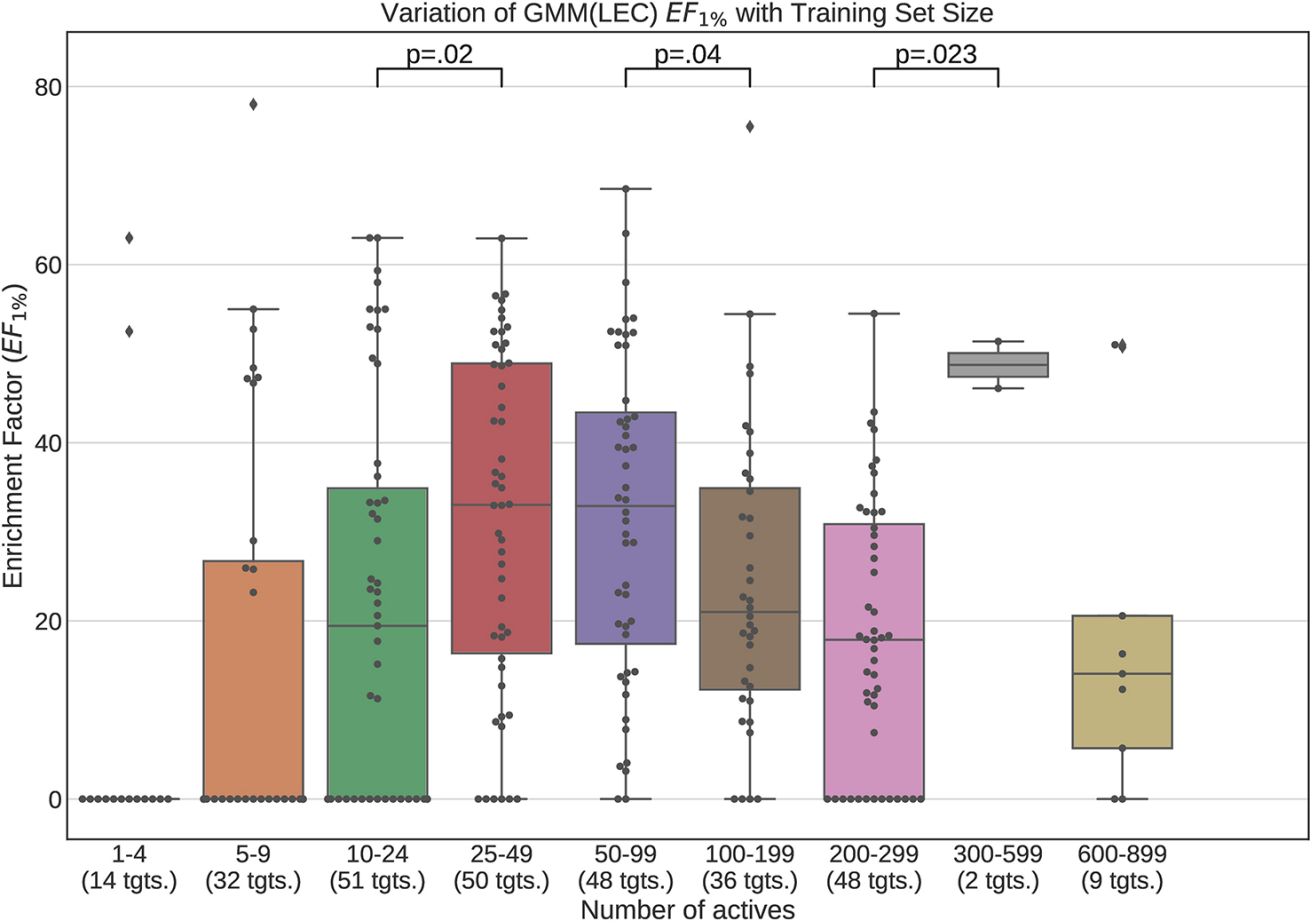
Performance variation of Lowest Energy Conformation (LEC) model Gaussian Mixture Model with number of actives. Scatter plot indicates one point per template within the given range. The number of templates captured within the range is indicated in the axis labels. Note that multiple points belonging to the same target could fall within a single range due to the binning thresholds used.

The statistical significance annotations were computed using the Wilcoxon rank-sum test (Mann and Whitney, 1947). This is a non-parametric test and therefore does not assume normality in the data. We have visually checked the distribution for each bin using histograms and found that they were not normal. It also assumed that the groups being compared are independent and not paired, which is the case with our box plots. The Wilcocon rank-sum test tests the null hypothesis that for any two observations *a* and *b* drawn from group *A* and group *B* respectively, the probability of *a* being greater that *b* is the same as that for *b* being greater that *a*. This test is used to investigate whether two sampling distributions are the same.

It is apparent from these figures that performance is better maintained for low number of actives by using full conformer models than by LECs. This is most pronounced for Neural Networks as well as GMMs, however it is also apparent for Isolation Forests, albeit more weakly. Nevertheless, even for small active training sets for which the mean performance is low, outliers are apparent with high enrichment factors. This shows that the performance of the methods we have explored is highly dependent on the protein target that is being considered and it is difficult to know *a-priori*, how well a method will perform given the number of available actives.

For LEC models a performance peak is apparent at around 25–49 actives, beyond which performance degrades again. We observed this effect on GMMs and Isolation Forest models, but not on Neural Networks. It is possible that implementing a more comprehensive parameter sweep during the tuning of these models could eliminate or reduce this effect. For example, in the case of GMMs, allowing a larger number of Gaussian components would probably resolve the active clusters better and improve performance for larger numbers of actives.

A general observation in our results is that, across the machine learning models that we trained, those trained on full conformers preserve good performance when trained with as little as 5–9 actives, while with those trained on LECs, the cutoff is in the 10–24 actives range. These results indicate that for small datasets, models should be trained using full conformer models.

### Running Times

In order to understand how the time required to train and perform a retrospective virtual screening run varies with dataset size, we plotted the time taken to perform our experiments against the corresponding dataset portion used as training set using box plots, with separate boxes representing the run-time for each machine learning algorithm. The timings include the time taken to train the final, tuned model and evaluate the molecules under test. This does not include the time required to generate the conformers and the USR and ElectroShape 5D descriptors. These plots can be found in the **Supplementary Material** (**Figures S16**–**S19**). Additionally we have also presented running-time statistics in **Table S1**.

Note that, if used in a prospective screening scenario, a machine learning model would have been pre-trained from the available training data, therefore the time required for training would not be a factor when measuring the running time for such a study. In this case, however, since a retrospective experiment was being carried out we considered the total time required for training as well as testing/evaluation to be an important consideration.

It is apparent from the plots supplied in the **Supplementary Material** that GMMs were the quickest models overall for LEC models (8s ± 11s mean time) and the second quickest for the full conformer models (787s ± 868s mean time). For full conformer-trained models, GMMs were quicker for dataset fractions up to 60% of the full dataset, however, were slower than Isolation Forest for dataset fractions larger than 60%. At the 30% fraction the GMM running time increased. This could have been caused by transient resource contention on the machine on which the experiments were being run.

Isolation Forest speed performance compared favourably to GMMs for large datasets when using full conformer models (397s ± 373s mean time for isolation forest vs. 787s for GMMs), however, for smaller datasets using LECs it was considerably slower than the other algorithms, including ANNs (453s ± 423s for Isolation Forest vs. 131s ± 89s for ANNs). This is quite surprising and is likely due to the fact that no matter the size of the training data, an ensemble of decision trees of comparable size need to be created by the algorithm. Tweaking the hyperparameters to use smaller ensembles for LECs would probably make this model faster, however, this was not attempted in this study.

Neural Networks appear to be the most consistent with respect to speed performance. In general, it is the slowest algorithm (1855s ± 1659s mean time for full conformers and 131s ± 89s mean time for LECs), except for Isolation Forest in the LEC scenario.

It is worth noting that, notwithstanding the necessity to train the machine learning models before running the virtual screening procedure, the total time required to perform our retrospective screening on each target took, on average, a much shorter time to complete than the standard USR algorithms which took, on average 10 times more time to complete. Part of this discrepancy is likely the efficiency of our Python implementation of USR, which must necessarily be slower than the C-based implementations of the algorithms in the scikit-learn library. The magnitude of the difference, however, makes it unlikely for this to be the entire explanation. A large part of the discrepancy also comes from the fact that, in USR, all the conformers in the test set of molecules must be compared to every conformer of every active template. Over the course of an entire retrospective screening cycle, this adds up to a large amount of computation.

With machine learning algorithms, however, this is not necessary. The bulk of the running-time when using machine learning methods is the training of the model, however this, in general, does not require the repeated comparison of all the data points with all the active data points in a Cartesian product fashion. Additionally, once a model is trained, classifying new data points is generally a fast process because it does not involve comparing the new point with the training data directly, but only requires that the new data be evaluated according to the model built during training. All this, clearly depending on which particular machine learning algorithm is being used, implies a much smaller amount of computation than the “brute force” approach inherent in standard USR.

## Discussion

Throughout this study we sought to answer two research questions, namely:

- Can machine learning techniques replace the naïve Manhattan distance in USR and USR-like methods to improve Virtual Screening performance?
- What is the minimal amount of data required to adequately train USR and USR-like machine learning models?

In pursuit of the first question, we used the datasets provided in DUD-E to generate a suitable number of conformers to adequately sample the conformational space of the molecules from which we generated corresponding USR and ElectroShape 5D descriptors.

We then selected three suitable machine learning algorithms, namely Gaussian Mixture Models, Isolation Forests, and Artificial Neural Networks and we trained and evaluated these models using the descriptors we had previously generated. In doing so, we obtained results that significantly outperformed USR as well as ElectroShape 5D when using both the full conformer models of the active molecules as training data, as well as when using only the Lowest Energy Conformations (LECs). Concretely, in terms of *EF*_1%_ the best mean improvement over ElectroShape 5D was that of 430% obtained using GMMs trained on full conformers, the same models having obtained a maximum improvement of 941% over ElectroShape 5D. This was followed by a mean improvement of 328% with a maximum of 636%, obtained by ANNs, again trained on full conformer models. When using LECs as training data, GMMs obtained a mean performance improvement of 291% and a maximum of 829%, outperforming ANNs with a hidden layer size of 100, which obtained a mean improvement of 256% with a maximum of 613%. It is clear, however, that some targets are more responsive to screening by USR descriptors, there being a relatively large variance in the mean performance figures. This is also reflected in the literature (Armstrong et al., 2009; Ballester et al., 2009; Armstrong et al., 2010; Armstrong et al., 2011) and is, therefore, expected.

These improvements over ElectroShape 5D are of a similar magnitude to the performance increase afforded by ElectroShape 5D itself over USR and are, therefore, highly significant. Machine learning algorithms assimilate the features of all the active molecules into a single model, in contrast to the naïve USR-based algorithms which can only consider one molecule at a time as a search query. This feature of machine-learning algorithms appears to make a large difference to the similarity matching performance in the LBVS context when compared with the standard algorithm for the USR family of methods.

In order to explore our second research question, we trained the machine learning models on progressively smaller fractions of the selected DUD-E targets so as to explore the manner in which the performance of the models varied whilst decreasing training dataset size. Our results demonstrate that when using full conformers to train the models, better performance is obtained when the number of actives is low. In general a performance peak is observed when training with 25–49 actives. With the LEC models, this peak is more pronounced, indicating that for small active training sets it is more advantageous to train with full conformers than LECs.

We also observed that performance of our models was preserved when only 5–9 actives are used for training when using full conformer models while, for the LEC-trained models, the performance remained acceptable down to the 10–24 actives level.

Taking into account all the results obtained, in terms of VS performance as well as running times, and we come to the conclusion that GMMs were, overall, the most efficient models that we tested, achieving excellent performance in the shortest time (except for the largest datasets; see **Figures S16** and **S17** in **Supplementary Information**) and while also exhibiting good stability with decreasing dataset size.

## Conclusion

To the best of our knowledge, this research project constitutes the first study to explore the viability of several machine learning algorithms in their application to LBVS using USR and USR-like descriptors.

We have demonstrated the utility of applying machine learning methods to the LBVS scenario when using USR-like descriptors, managing to obtain significant performance improvements over both the USR and the ElectroShape 5D algorithms using the Gaussian Mixture Model (GMM), Isolation Forest and Artificial Neural Network (ANN) algorithms. The GMM models were found to achieve the best performance improvement over ElectroShape 5D in terms of enrichment factor, giving an improvement of 291% for LEC-trained models and 430% for full conformer trained models with maximum improvements of 829% and 940%, respectively. These results clearly represent non-trivial improvements over the classical, non-machine learning, USR family of methods.

Furthermore we demonstrated that these trained models maintain stable performance when trained with drastically smaller quantities of training data, especially when full conformer molecule models are used, maintaining statistically similar performance from full dataset down to the 5–9 active range for full conformer models.

We also demonstrated the significant advantages in terms of running times, where retrospective screening took, on average 10 times less time to complete using our machine learning models than for USR and ElectroShape 5D.

Due to the sheer magnitude of the options available when it comes to machine learning methods, this work must be considered as a starting point for further research into the topic of machine learning on USR, however, we believe that it makes a valid contribution to the field, as it demonstrates significant performance improvements over current state-of-the-art methods that do not use machine learning.

## Data Availability Statement

The datasets analyzed for this study can be found in the Database For Useful Decoys-Enhanced (DUD-E).

## Author Contributions

J-PE contributed to the conception and design of the study and guided and supervised the research. EB implemented and carried out the experiments and drafted the manuscript. J-PE revised and submitted the manuscript. All the authors have read and approved the final version of the manuscript.

## Funding

This research was partially funded by the Research Support Services Directorate at the University of Malta (Grant number MMERP04-18), as well as Amazon Web Services through their AWS Educate initiative. These funders were not involved in the study design, collection, analysis, interpretation of data, the writing of this article, or the decision to submit it for publication.

## Conflict of Interest

The authors declare that the research was conducted in the absence of any commercial or financial relationships that could be construed as a potential conflict of interest.

## Footnotes

1 https://github.com/pandegroup/vs-utils [Last Accessed 4 May 2019]

2 https://github.com/mcs07/MolVS [Last Accessed: 5 August 2019]

## Abbreviations

ANN, artificial neutral Nework; AUC, area under curve; CSR, chiral shape recognition; DG, distance geometry; DUD, directory of useful decoys; DUD-E, directory of useful decoys-enchanced; EF, enrichment factor; ETKDG, experimental-torsion knowledge distance geometry; GMM, gaussian mixture model; HTS, high throughput screening; LBVS, ligand-based virtual screening; LEC, lowest energy conformation; ROC, receiver operator characteristic; SBVS, structure-based virtual screening; SMILES, simplified molecular input line entry specification; USR, ultrafast shape recognition; USRCAT, ultrafast shape recognition with CREDO atom types; VS, virtual screening.
