# Supplementary material for: Applying Machine Learning to Ultrafast Shape Recognition in Ligand-Based Virtual Screening
Source: Front Pharmacol. 2020 Feb 19;10:1675. doi: 10.3389/fphar.2019.01675 (PMC7042174; doi:10.3389/fphar.2019.01675)
Supplement: Supplementary file 1 [file DataSheet_1.pdf]

Supplementary Material

1 SUPPLEMENTARY DATA

1.1 Figures

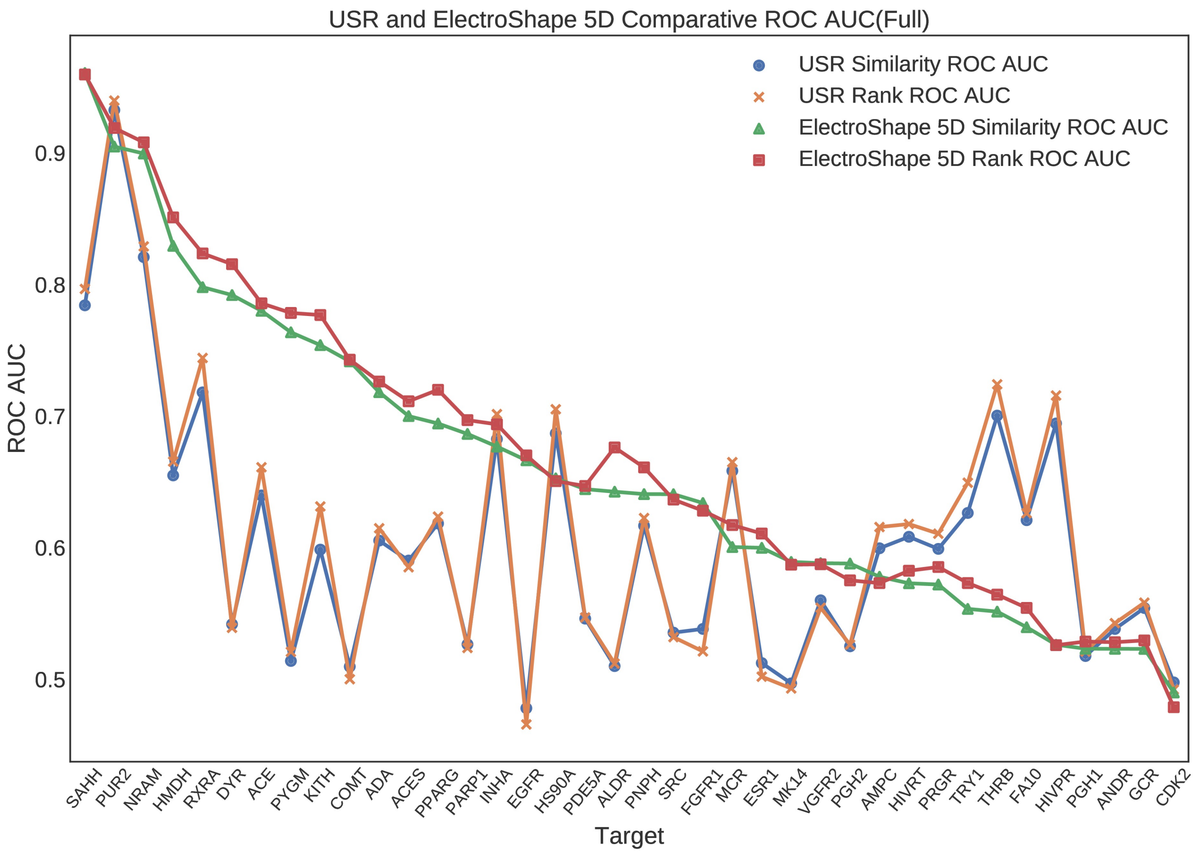

Figure S1. USR and ElectroShape 5D comparative AUC performance obtained from retrospective screening using full conformer model.

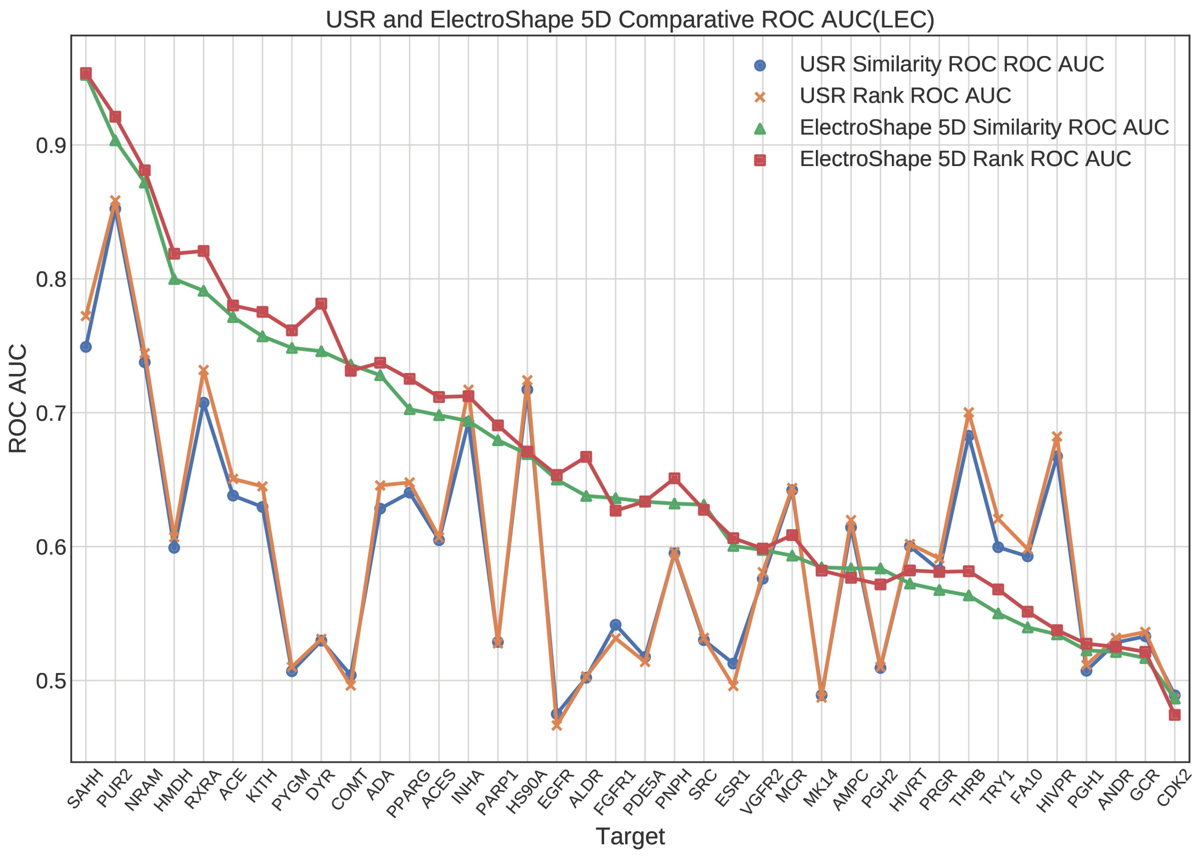

Figure S2. USR and ElectroShape 5D comparative AUC performance using Lowest Energy Conformers.

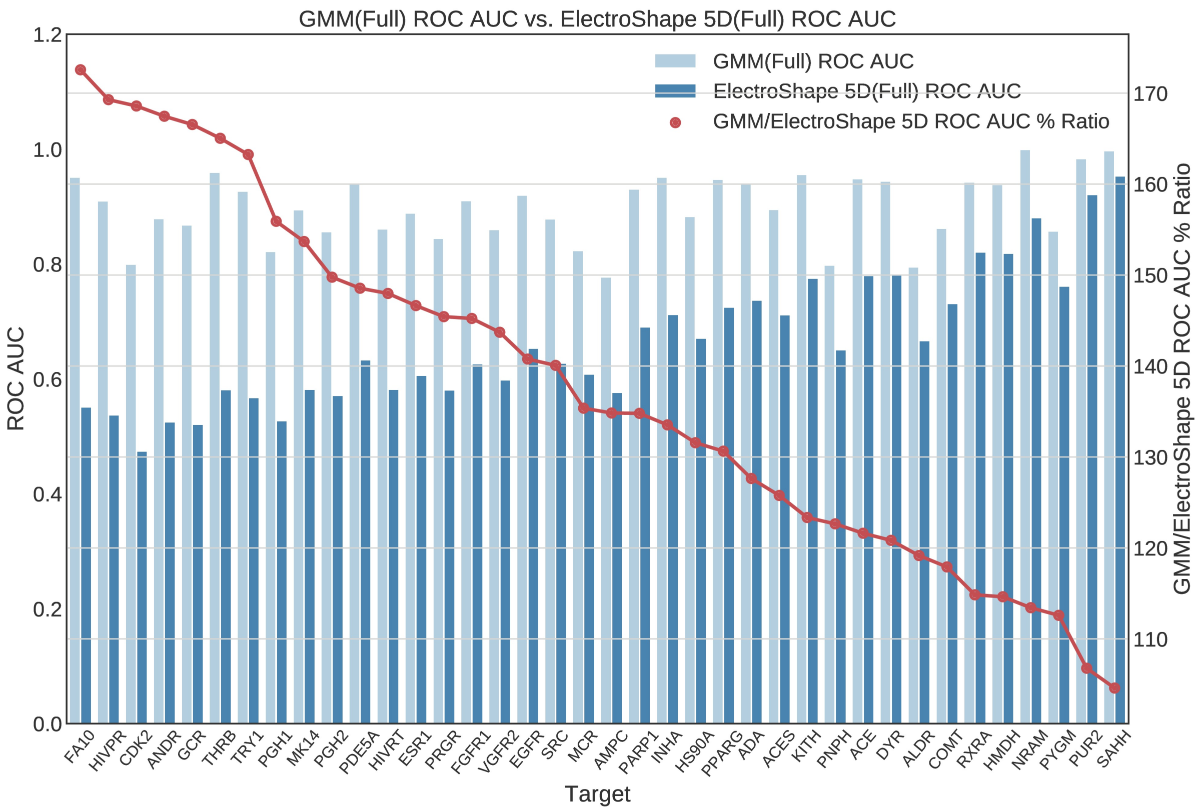

Figure S3. Comparative AUC of GMM vs.ElectroShape 5D using full conformer model. Mean improvement 138%. Maximum improvement 173%

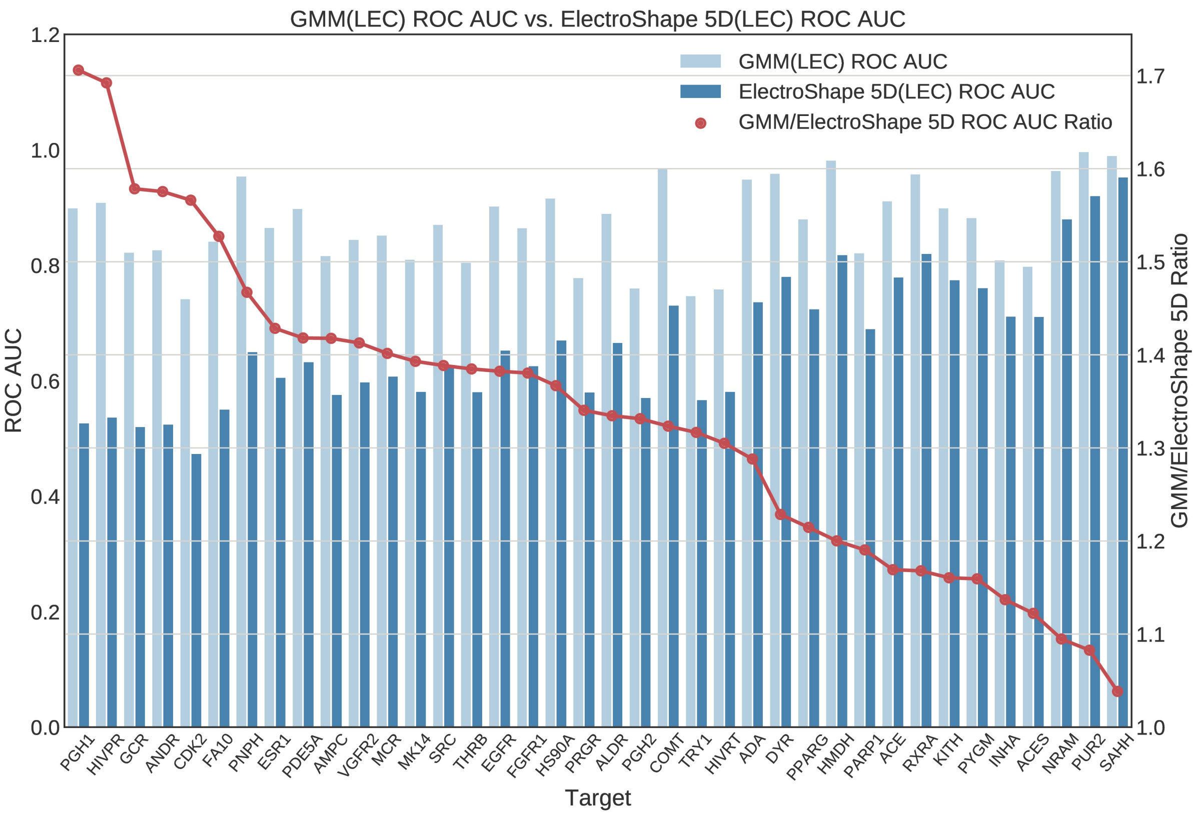

Figure S4. Comparative AUC of GMM vs ElectroShape 5D using full conformer model. Mean improvement 133%. Maximum improvement 171%.

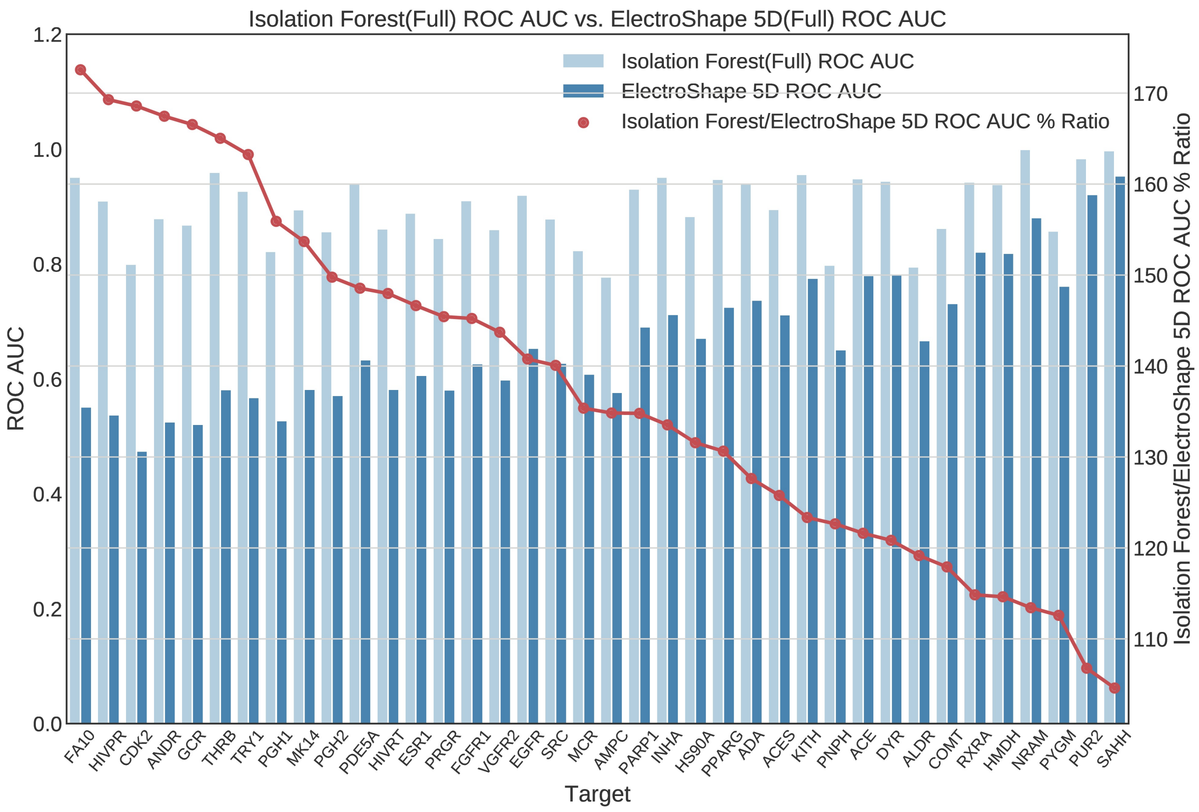

Figure S5. Comparative AUC of Isolation Forest vs.ElectroShape 5D using full conformer models. Mean improvement 123%. Maximum improvement 152%.

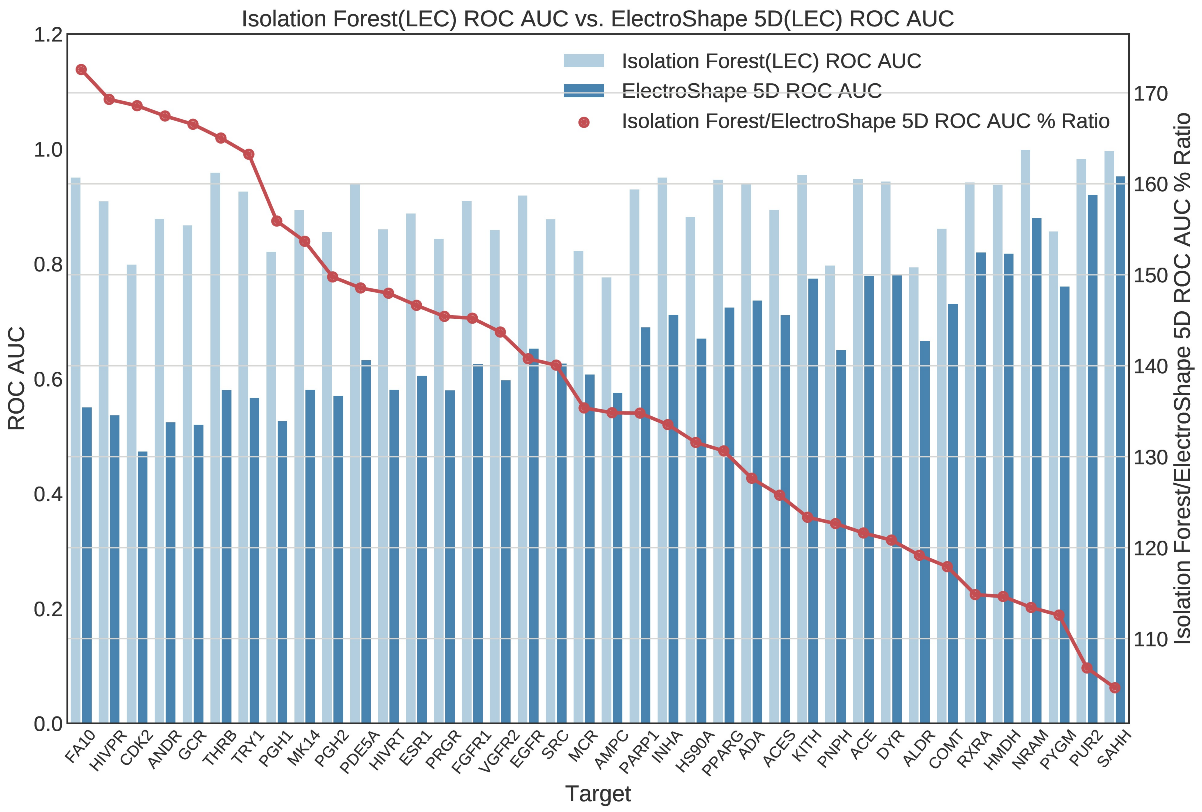

Figure S6. Comparative AUC of Isolation Forest vs ElectroShape 5D using full conformer model. Mean improvement 126%. Maximum improvement 155%

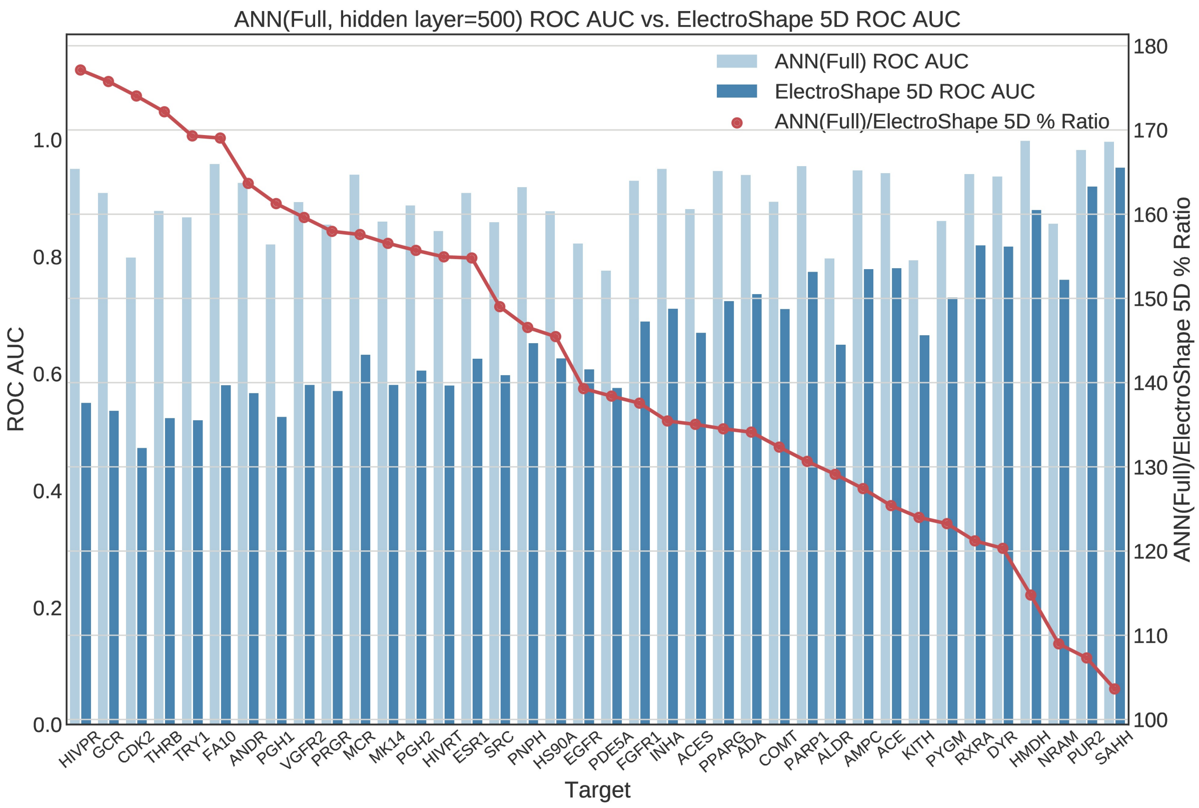

Figure S7. Comparative AUC of Neural Network vs. ElectroShape 5D using full conformer model(hidden layer size=500). Mean improvement 143%. Maximum improvement 177%.

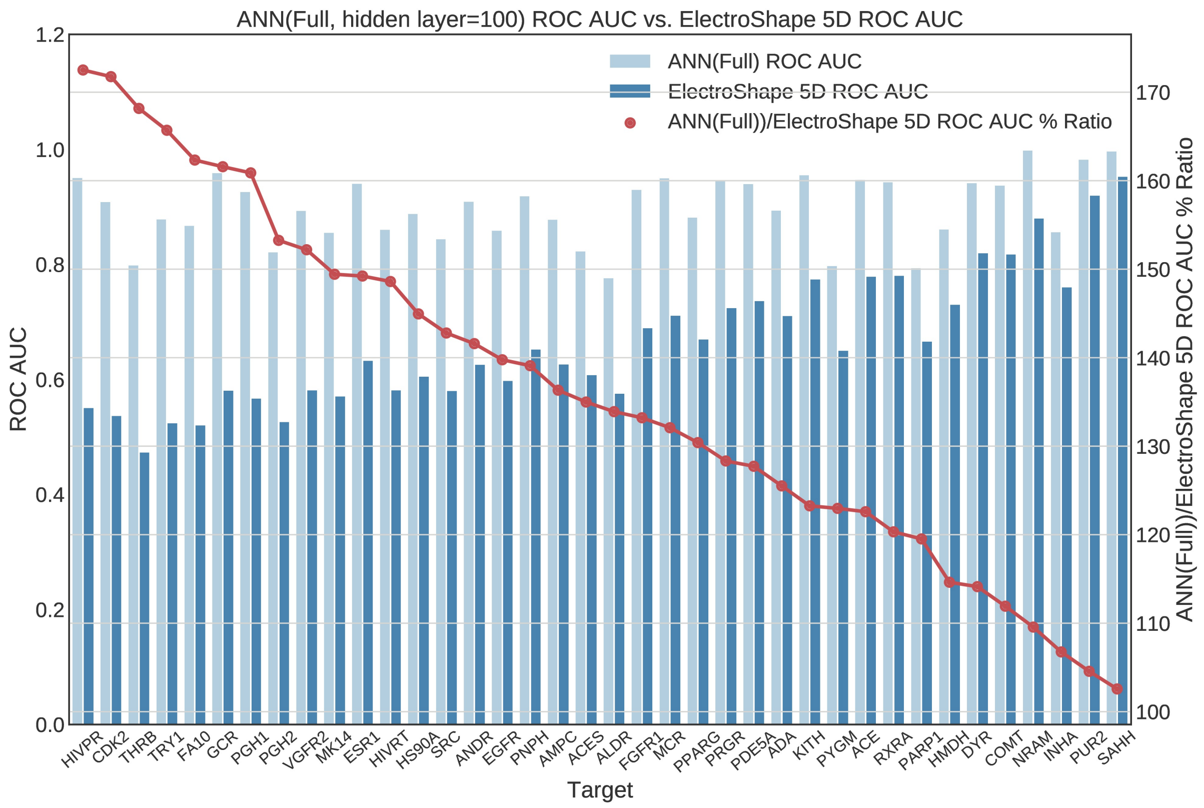

Figure S8. Comparative AUC of Neural Network vs. ElectroShape 5D using full conformer model(hidden layer size=100). Mean improvement 136%. Maximum improvement 173%.

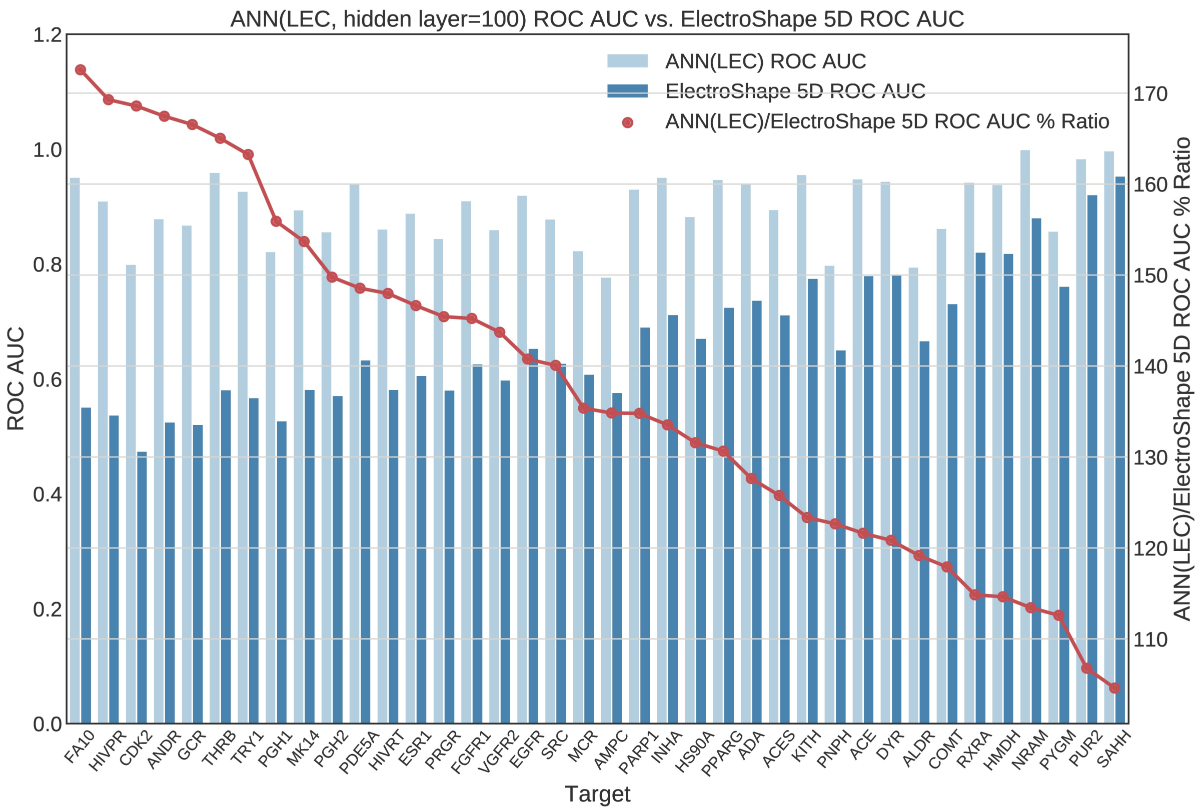

Figure S9. Comparative AUC of Neural Network vs. ElectroShape 5D using LECs(hidden layer size=100). Mean improvement 139%. Maximum improvement 175%

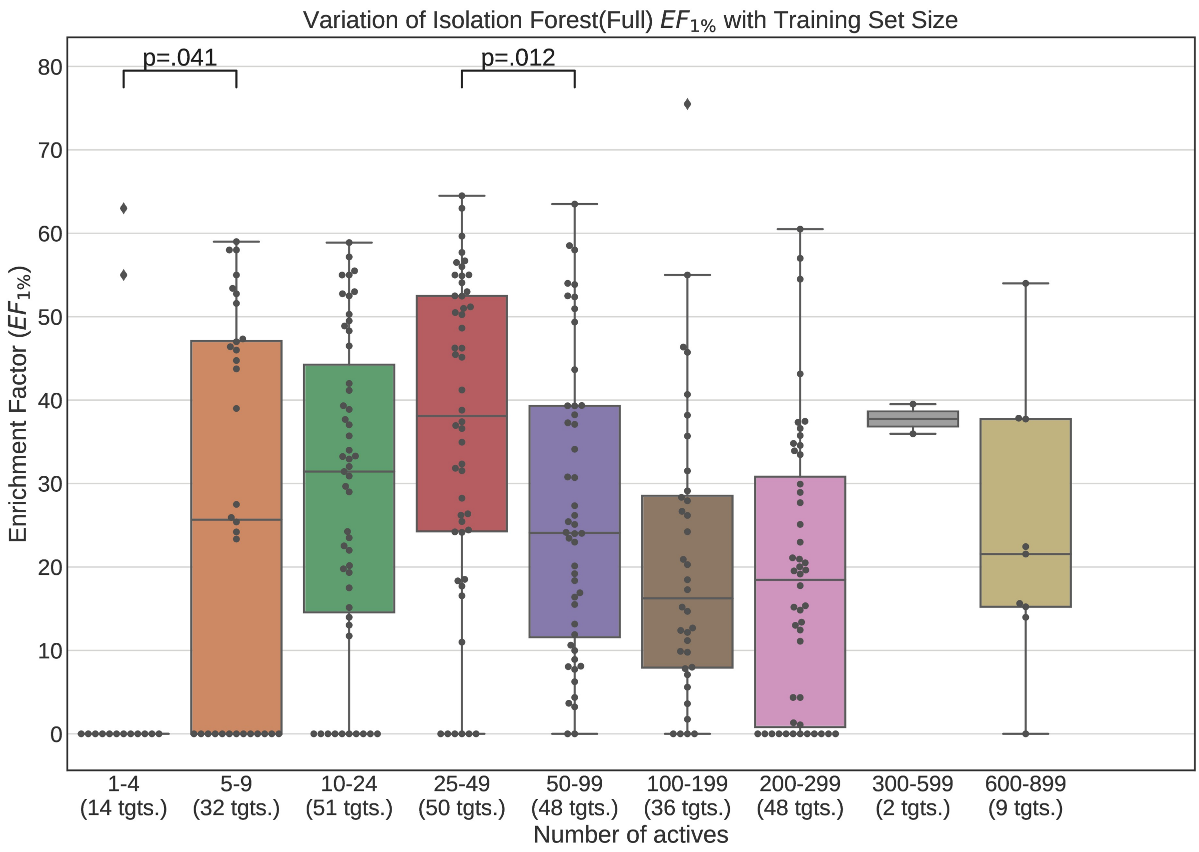

Figure S10. Performance variation of full-conformer model Isolation Forest with number of actives.

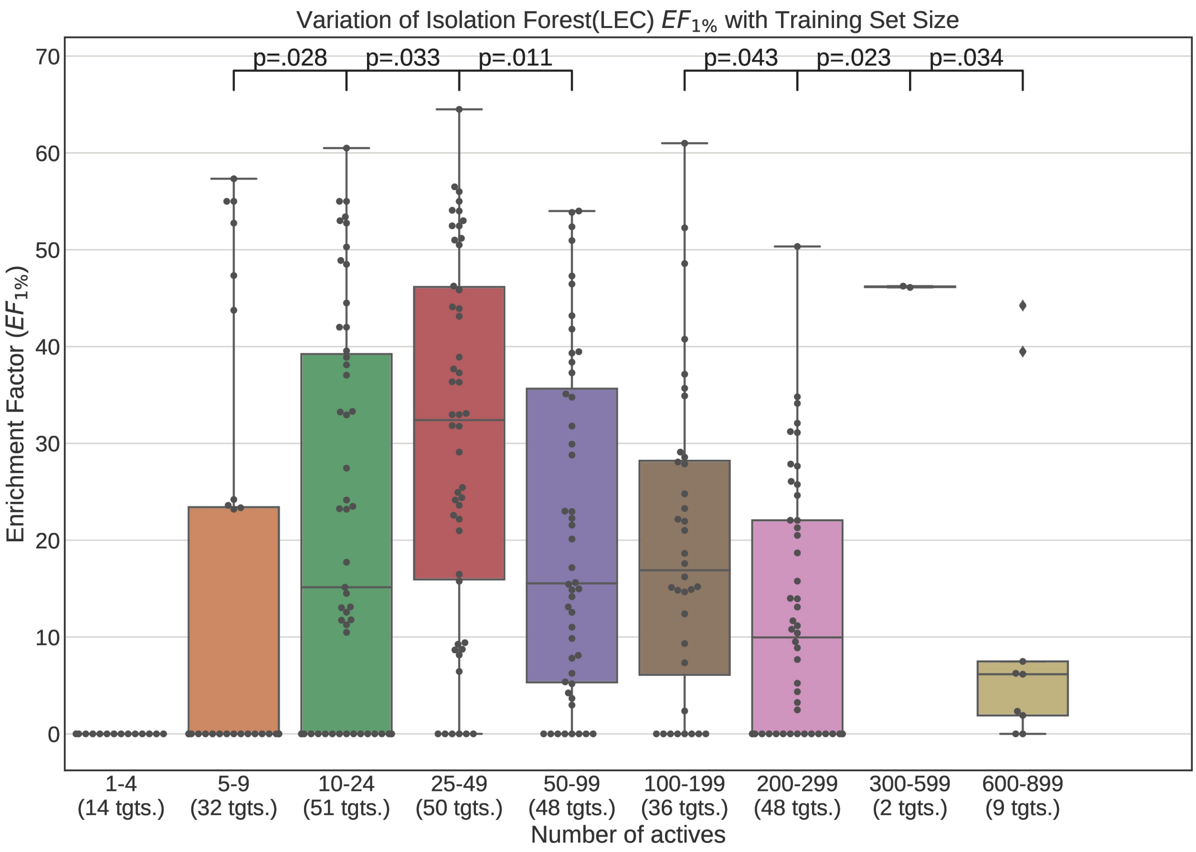

Figure S11. Performance variation of LEC model Isolation Forest with number of actives.

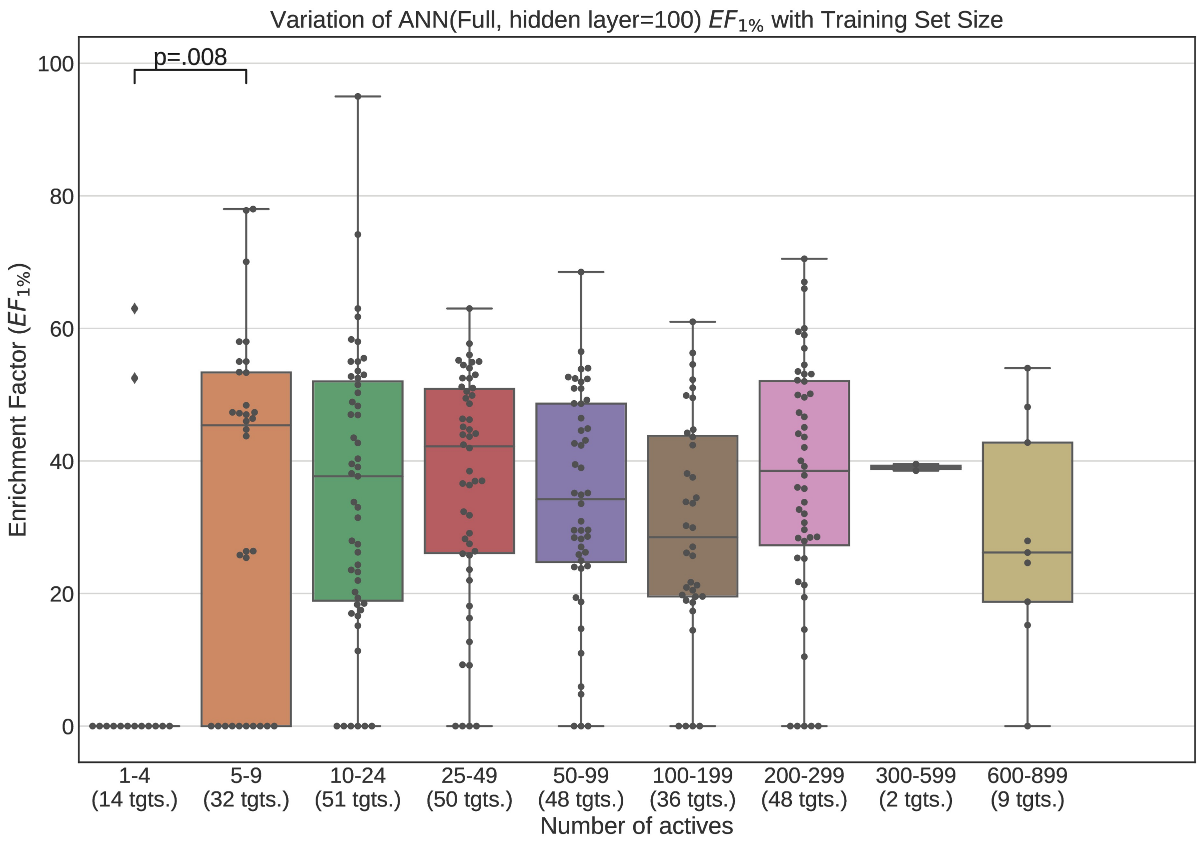

Figure S12. Performance variation of full-conformer model neural network (hidden layer size=100) with number of actives.

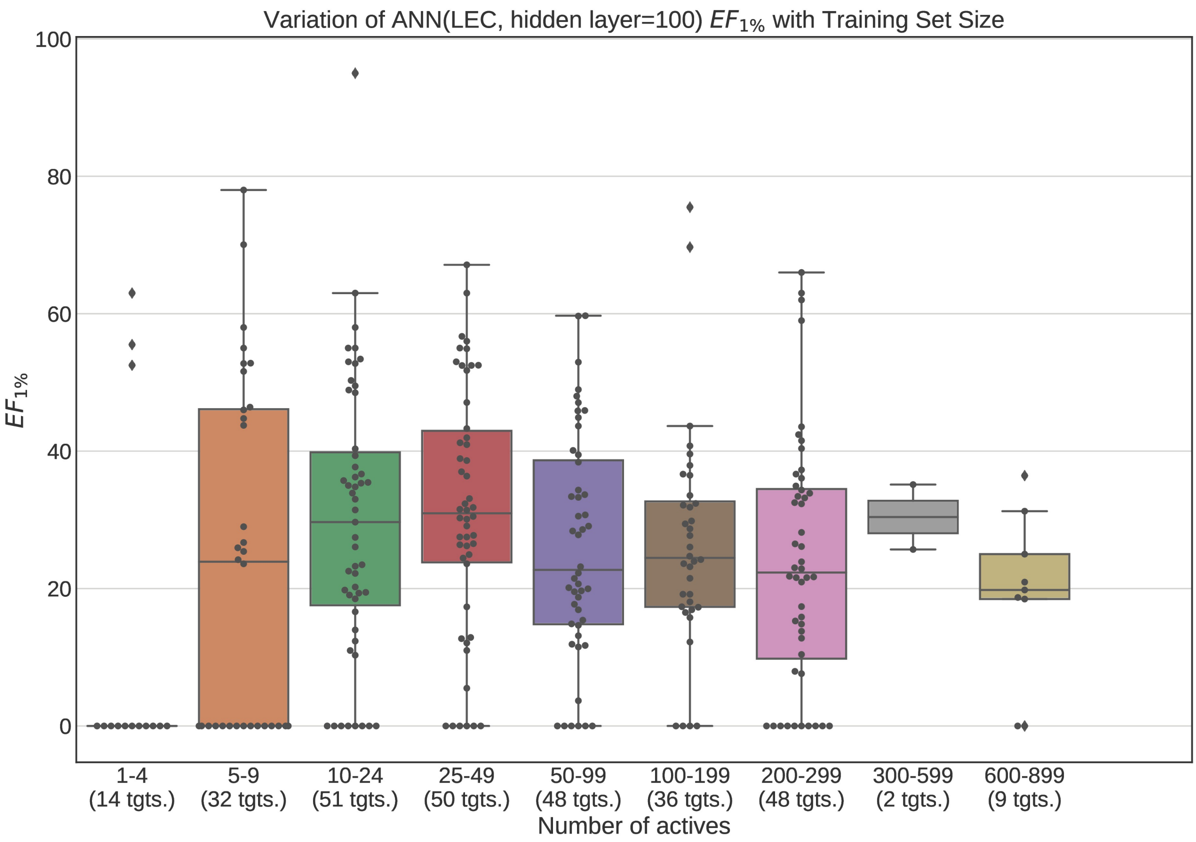

Figure S13. Performance variation of LEC model neural network (hidden layer size=100) with number of actives.

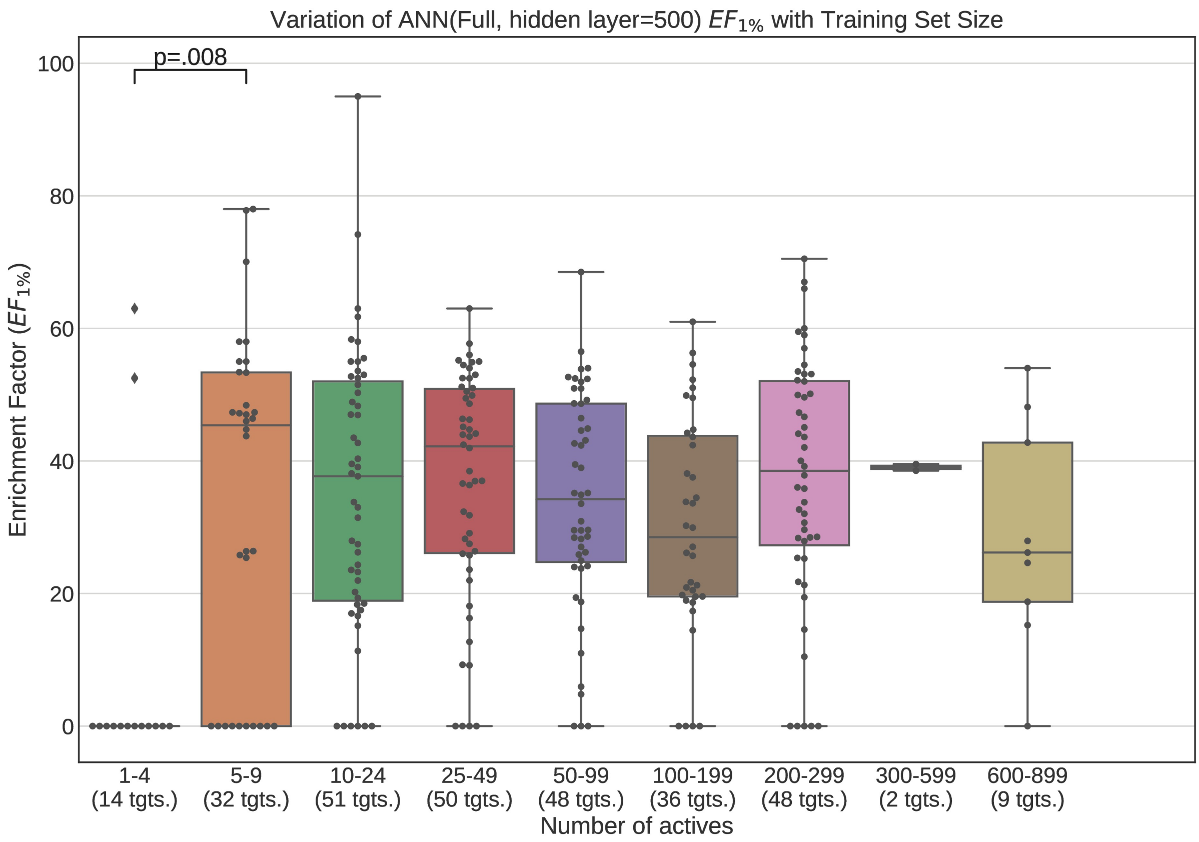

Figure S14. Performance variation of full-conformer model neural network (hidden layer size=500) with number of actives.

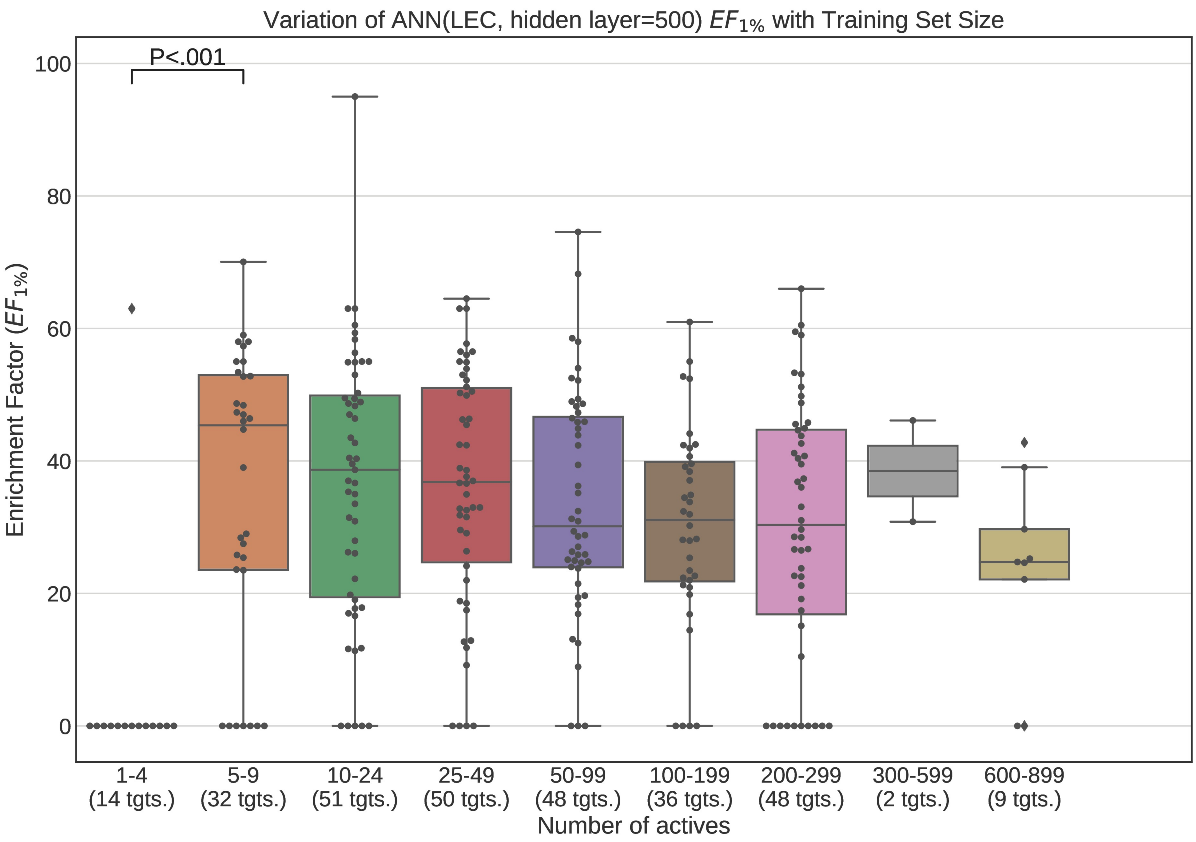

Figure S15. Performance variation of LEC model neural network (hidden layer size=500) with number of actives.

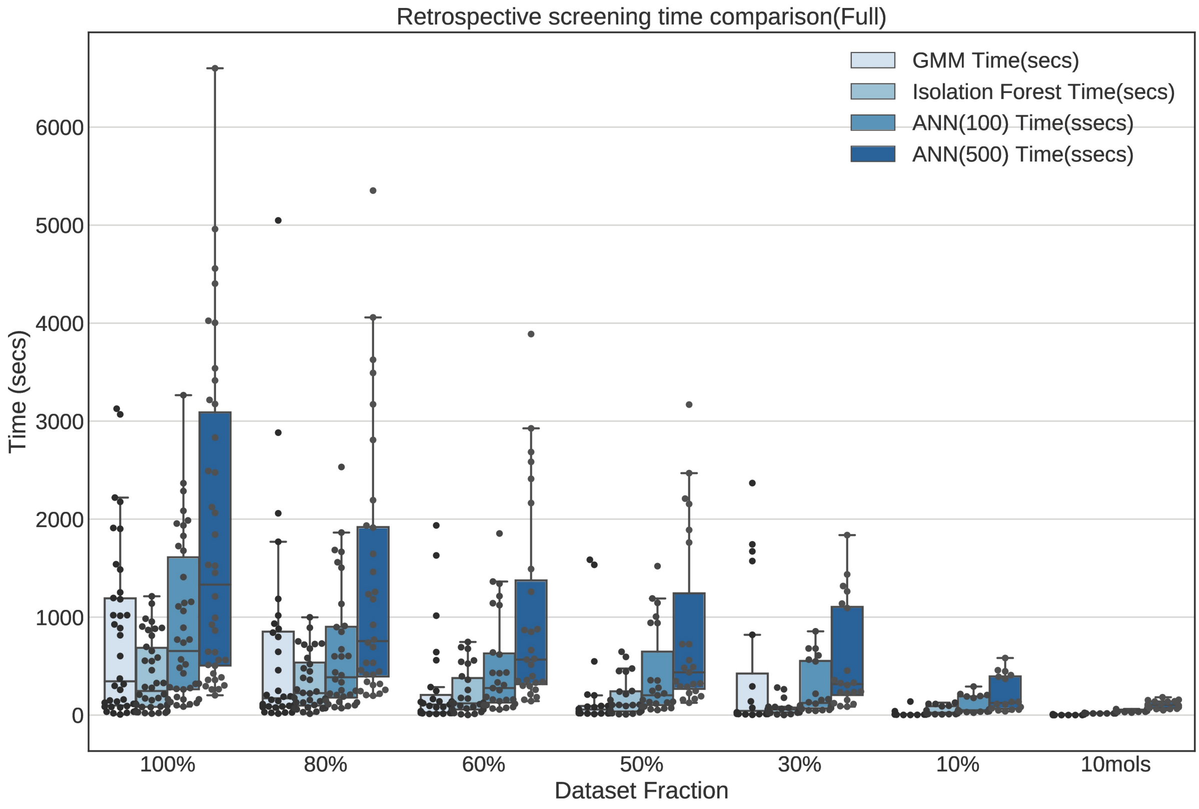

Figure S16. Run-time for training and retrospective screening for full conformer models.

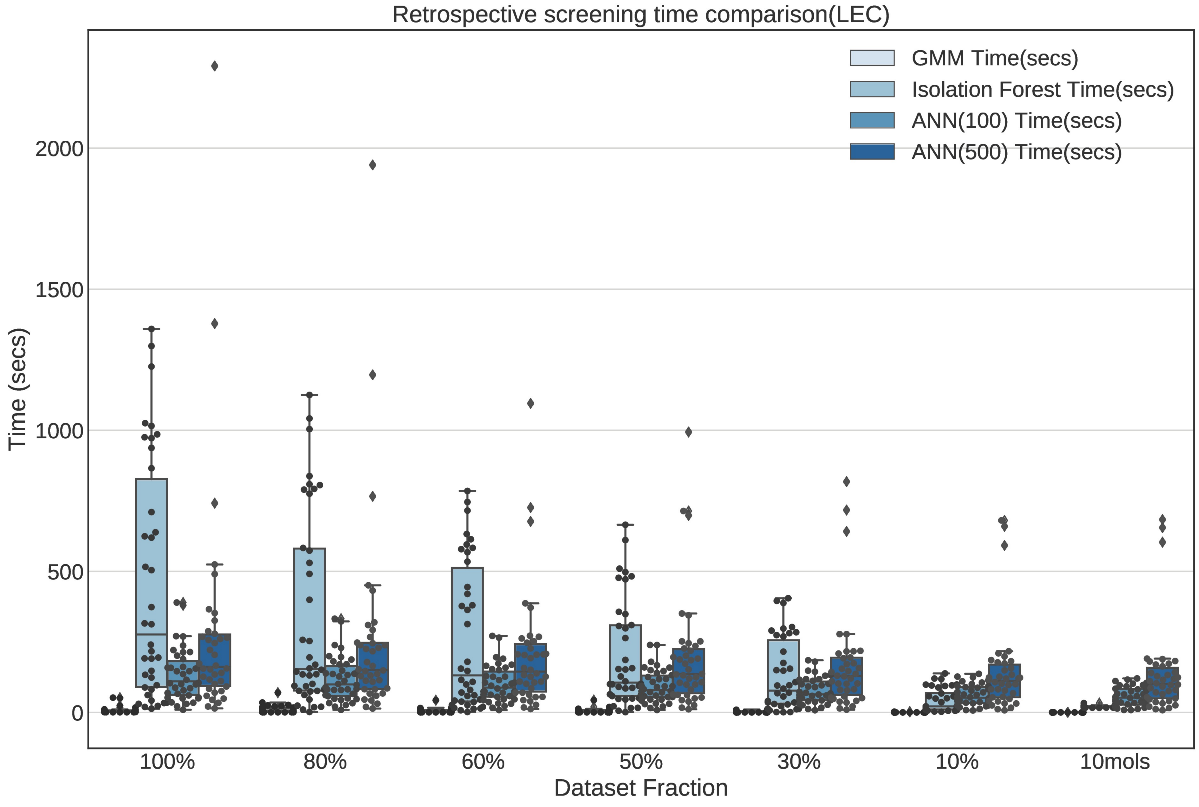

Figure S17. Run-time for training and retrospective screening forLEC models.

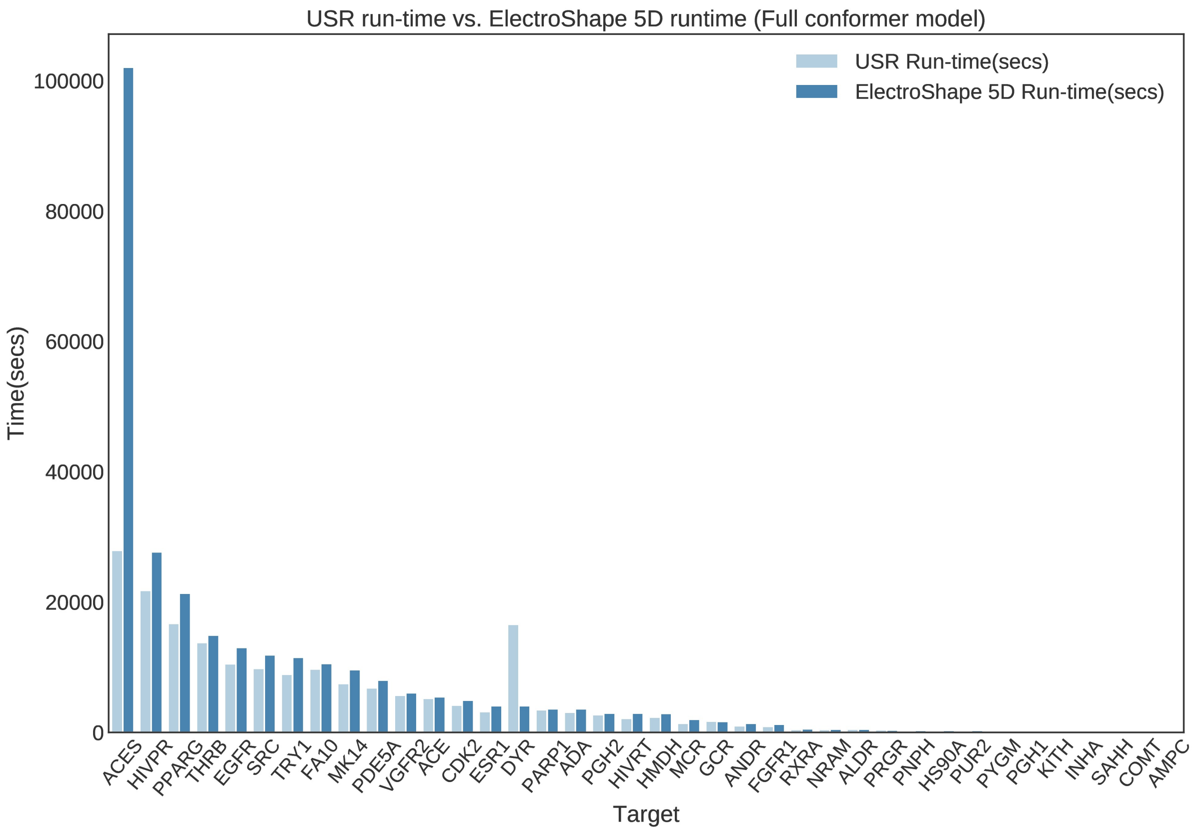

Figure S18. Run-time in seconds for USR and ElectroShape 5D retrospective screening using full conformer models.

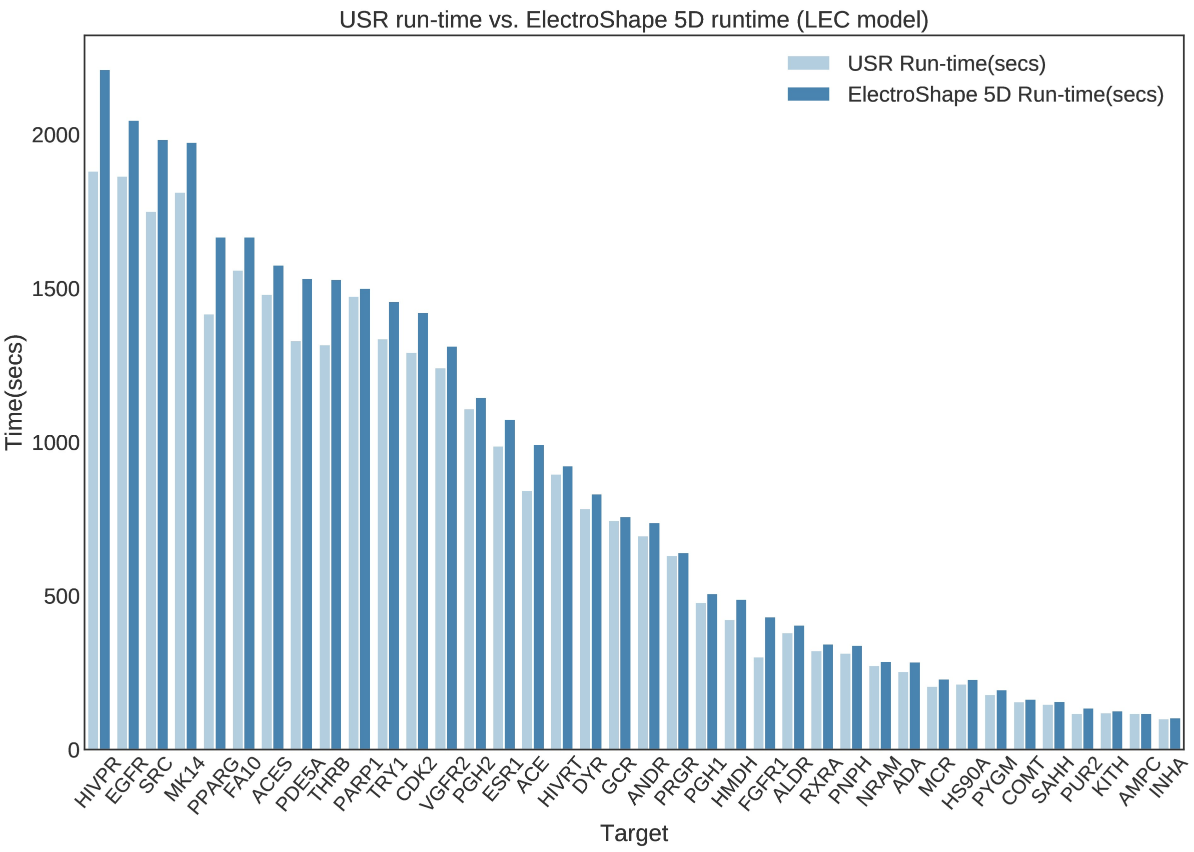

Figure S19. Run-time in seconds for USR and ElectroShape 5D retrospective screening using LECs.

1.2 Tables

LEC	USR	ES5	GMM	Isolation Forest	ANN(100)	ANN(500)
Mean(s) (±s.d.)	804(±594)	883(±661)	8(±11)	453(±423)	134(±92)	285(±413)
Max(s)	1,882	2,212	52	1,359.00	390	2,290
Min(s)	100	104	0.7	13	10	14
**Full Conformers**	**USR**	**ES5**	**GMM**	**Isolation Forest**	**ANN(100)**	**ANN(500)**
Mean(s) (±s.d.)	5021(±6699)	7409(±16985)	789(±868)	397(±373)	934(±825)	1855(±1649)
Max(s)	27,940	102,034	3,126	1,212	3,264	6,600
Min(s)	59	61	6	14	87	202

Table S1. Tabulated runnimg-time statistics for all LEC and full-conformer models. Timings are shown in seconds and include training and testing.

Target	Conformers (.sdf)	USR Descriptors	ElectroShape 4D Descriptors	ElectroShape 5D Descriptors
ACE	5.9G	336M	419M	493M
ACES	12G	567M	706M	834M
ADA	1.5G	90M	111M	131M
ALDR	1.5G	106M	128M	151M
AMPC	344M	29M	35M	41M
ANDR	2.4G	156M	190M	223M
CDK2	6.6G	375M	461M	542M
COMT	557M	42M	51M	60M
DYR	4.1G	240M	295M	345M
EGFR	14G	634M	791M	933M
ESR1	6.4G	326M	403M	475M
FA10	12G	547M	684M	807M
FGFR1	3.0G	143M	178M	209M
GCR	3.2G	181M	223M	262M
HIVPR	19G	888M	1.1G	1.3G
HIVRT	3.7G	232M	285M	335M
HMDH	4.4G	216M	271M	320M
HS90A	1.2G	65M	80M	94M
INHA	536M	33M	41M	48M
KITH	734M	41M	51M	60M
MCR	1001M	60M	74M	87M
MK14	12G	559M	695M	820M
NRAM	1.4G	89M	110M	129M
PARP1	5.4G	349M	423M	498M
PDE5A	10G	494M	617M	729M
PGH1	1.7G	117M	142M	168M
PGH2	4.3G	269M	328M	388M
PNPH	1000M	81M	98M	114M
PPARG	14G	656M	826M	976M
PRGR	2.5G	166M	202M	237M
PUR2	968M	53M	66M	78M
PYGM	972M	58M	71M	84M
RXRA	1.5G	88M	109M	128M
SAHH	412M	35M	42M	49M
SRC	13G	610M	762M	898M
THRB	12G	561M	702M	828M
TRY1	11G	512M	638M	751M
VGFR2	8.2G	404M	503M	592M

Table S2. The sizes on disk of the datasets generated/used in our experiments. Here is can be seen that the space required to store the 3D conformer data generated from the 2D SMILES representations of the compount datasets is orders of magnitude more than that required for the much condensed USR family of descriptors.
